# Research on government subsidy strategies for new drug R&D considering spillover effects

**DOI:** 10.1371/journal.pone.0262655

**Published:** 2022-02-10

**Authors:** Zhe Huang, Yipeng Lan, Xiangqi Zha

**Affiliations:** School of Business Administration, Shenyang Pharmaceutical University, Shenyang, Liaoning, China; Institute for Advanced Sustainability Studies, GERMANY

## Abstract

This paper studies the pharmaceutical production supply chain system composed of upstream and downstream pharmaceutical enterprises, and explores the impact of government subsidy strategies on the new drug research and development (R&D) decision variables and profits of pharmaceutical enterprises as well as social welfare, when considering both the horizontal spillover effects within the industry and the vertical spillover effects between industries. Comparing and analyzing the impact of these strategies including non-government subsidy strategy, pharmaceutical enterprise innovation input subsidy strategy, pharmaceutical enterprise innovative product subsidy strategy, patient price subsidy strategy, and patient medical insurance subsidy strategy. By establishing a four-stage Cournot duopoly model incorporating spillover effects, the equilibrium solutions are obtained by backward induction, and the impact of spillover effects on decision variables is investigated accordingly. Studies have shown that: (a) Government subsidy strategies can promote pharmaceutical enterprises’ R&D investment and have a positive incentive effect on the pharmaceutical enterprises’ profits and social welfare. (b) The patient medical insurance subsidy strategy is the optimal subsidy strategy, which can generate higher profits for pharmaceutical enterprises and higher social welfare. (c) The horizontal and vertical spillover effects have different effect on decision variables, enterprises’ profits and social welfare under various subsidy strategies.

## 1 Introduction

New drug had been defined as drug that has not been marketed within or outside China, according to the "Pharmaceutical Administration Law of the People’s Republic of China" (2019 version) and the "Drug Registration Regulation of China" (2020 version). Due to changes in specifications, dosage forms, and routes of administration, the drug has new active ingredients, chemical structures or changes in their pharmacological effects, which can be verified and marketed by the Drug Evaluation Center [1, 2]. New drug research and development (R&D) and listing are related to the life and health of patients. New drug R&D has the characteristics of high investment, high risk, long cycle, strong spillover effects, and high dependence on intellectual property rights. In particular, spillover effects will affect the innovation activities of pharmaceutical enterprises [3, 4].

Spillover effect has been defined as effect that when an organization conducts an activity, it will not only produce the expected effect of the activity, but also affect other organizations or society outside the organization. It mainly includes knowledge spillover effect, technology spillover effect and economic spillover effect [5, 6]. At present, enterprises and scientific researchers have made great breakthroughs in technological innovation and product R&D based on spillover effects. There are obvious spillover effects in new drug R&D of pharmaceutical industry. New drug R&D can not only indirectly reduce the unit drug production cost, but also reduce the innovation cost of other links by the spillover effects [7]. Therefore, it is an important and practical research topic that how pharmaceutical enterprises carry out new drug R&D activities based on spillover effects. In order to encourage innovation, Chinese government has issued documents such as the "Outline of National Innovation-Driven Development Strategy" and "Pharmaceutical Industry Development Planning Guide", established special funds and formulated subsidy strategies to promote new drug R&D [8]. The government subsidy strategies are mainly divided into innovation input subsidy strategy and innovative product subsidy strategy according to the product innovation and process innovation of enterprises, or divided into manufacturer subsidy strategy and consumer subsidy strategy according to the subsidy objects. Different subsidy strategies affect the social demand of products, the innovation input and output level of enterprises [9]. Therefore, in the pharmaceutical industry, research on the impact of different government subsidy strategies on new drug R&D is particularly important. Based on these, it has both great theoretical significance and good practical application value to research on the government subsidy strategies for new drug R&D considering spillover effects.

We focus on the research on the government subsidy strategies for new drug R&D considering spillover effects and attempt to address the following questions:

- How does the different government subsidy strategies affect the innovation input, the price of products, the profits of pharmaceutical enterprises and the social welfare.
- How does the horizontal and vertical spillover effects affect the decision variables of pharmaceutical enterprises and the social welfare under the different government subsidy strategies.
- What is the optimal government subsidy strategy under the horizontal and vertical spillover effects?

To answer these questions, firstly, according to the background of new drug R&D and government subsidy objects and strategy modes, three models will be established, including non-government subsidy strategy, pharmaceutical enterprise subsidy strategy and patient subsidy strategy. The pharmaceutical enterprise subsidy strategy is divided into innovation input subsidy and innovative product subsidy; the patient subsidy strategy is divided into price subsidy and medical insurance subsidy. Then, considering the horizontal and vertical spillover effects among new drug R&D enterprises, the equilibrium output of enterprises, innovation input, product price, enterprises’ profits and social welfare are solved under different models. Finally, based on the above researches, the optimal strategies under different government subsidy strategies are compared and analyzed, providing decision-making reference for the government to formulate appropriate subsidy strategies.

The rest of this paper is organized as follows: the Section 2 is literature review, the Section 3 is symbol definition and problem description, the Section 4 is basic model, the Section 5 is comparison of equilibrium solutions of five models, the Section 6 is numerical analysis, the Section 7 is conclusion and policy implications and provides suggestions for future research.

## 2 Literature review

At present, few researches have been done on the government subsidy strategies of new drug R&D considering spillover effects. However, there are some researches focus on spillover effects of R&D activities and government subsidy strategies [10–38].

In terms of the spillover effects of R&D activities, scholars have found that spillover effects are one of the important factors that affect the decision-making of R&D activities of enterprises. Spillover effects can be divided into two types: horizontal spillover effects and vertical spillover effects. Appropriate spillover effects help promote enterprises’ R&D activities. To explore the relationship between spillover effects and R&D decisions by constructing decision-making models is one of the areas where scholars focus on research [10–17]. For example, Liu, et al. [10] established a differential game model to investigate the impact of spillover effects under the different innovative drug R&D strategies of pharmaceutical firms; Xu, et al. [11] established a two-stage game model to discuss the impact of technology spillovers on enterprise R&D investment; Li [12] focused on the issue of supply chain carbon emission reduction, analyzed the vertical spillover effects of green technology R&D activities on the impact of emission reduction decision variables and profits of enterprises; Huang, et al. [13] established a two-stage game model and discussed the optimal spillover level and the optimal innovation R&D strategy of pharmaceutical enterprises under the conditions of both internal and inter-company spillovers; Lin [14] studied a game theoretical model that captures the interactions among three parties, and then demonstrated that knowledge spillovers have a significant impact on the eco-innovation levels, the economic benefits, and the total social welfare of supply chain; Wang [15] comprehensively considered the double marginal effect of innovation and horizontal technology spillover effect between supply chain partners, and analyzed the influence of two modes of non-cooperative R&D and cooperative R&D on innovation activities of enterprises; Sun, et al. [16] focused on cooperative R&D, analyzed the impact of different R&D models on R&D investment between upstream and downstream enterprises under the conditions of horizontal and vertical spillovers; Yu [17] established a three-stage game model and analyzed the impact of environmental policy on green technological innovation of enterprises in the presence of technology spillover effect.

In terms of government subsidy strategy researches, cholars have discussed the government subsidy forms in different industries, and explored the influence of different subsidy forms on industry research and development activities [18–25]. For example, Li, et al. [18] established a game model to analyze the impact of technology spillover rate, pollution damage degree and pollution tax on the choice of enterprises’ pollution control technology under the conditions of non-government subsidy and optimal government subsidy; Cao [19] focused on the decision-making problem of green efforts in the supply chain, discussed the impact of three government subsidy strategies, including non-government subsidy, manufacturer subsidy and consumer subsidy on the green efforts of manufacturers and retailers; Yao [20] established duopoly competition model and studied the impact of green subsidy and spillover effects on the behavior of government and enterprises; Deng [21] based on the perspective of product quality improvement innovation, established a three-stage dynamic game model and discussed enterprise R&D investment decision and government optimal subsidy policy under different R&D models; Sheng [22] established an evolutionary game model on the premise that the company’s products have substitutability and the production costs are different, and analyzed the influence of government subsidy on the selection of enterprise innovation models; Chen [23] used biosimilars as an example and studied the impact of different government subsidy strategies on enterprise innovation; Fan [24] took the investment spillovers and technological risks into consideration, and analyzed the R&D and production strategies of enterprises under different conditions; Yang, et al. [25] established a dynamic game model between enterprises and the government in an oligopoly market and analyzed the optimal production and environmental R&D decisions of enterprises and the government’s optimal subsidy policy.

Existing researches on spillover effects of R&D activities point out that the spillover effects of R&D activities can be divided into horizontal spillover effects and vertical spillover effects, it also provide the calculation methods of decision variables such as balanced output, profit, innovation input and social welfare [10–17, 26–31]. Researches on government subsidy strategies are mainly applied in the field of green innovation. They analyzed and compared various subsidy strategies from different subsidy objects such as manufacturer subsidies and consumer subsidies, or from different subsidy modes such as innovation input subsidies and innovative product subsidies [18–25, 32–38]. All of these provide theoretical and methodological support for the study on government subsidy strategies of new drug R&D considering double spillover effects. However, previous researches have hardly considered horizontal spillover effects, vertical spillover effects, manufacturer subsidies and consumer subsidies at the same time, and rarely analyzed new drug R&D decision-making issues. Based on these, this paper proposes a decision-making method of government subsidies for new drug R&D based on the government perspective, which comprehensively considers the vertical spillover effects and the horizontal spillover effects.

## 3 Symbol definition and problem description

### 3.1 Symbol definition

The symbols used in the model building process of this article are shown in Table 1.

**Table 1:** Symbol definitions.

Symbol	Definitions
*Q*	Output of products produced by pharmaceutical enterprises.
$c_{1}$	Cost per unit of intermediate product produced by upstream pharmaceutical enterprises.
$c_{2}$	The cost of downstream pharmaceutical enterprises converting intermediate product into a final product.
*w*	Upstream pharmaceutical enterprises’ pricing of each unit of intermediate products.
*p*	Downstream pharmaceutical enterprises’ pricing for each unit of finished product.
*a*	market capacity.
*b*	Consumers’ sensitivity to price.
*x*_*si*_	R&D output of upstream pharmaceutical enterprises.
*x*_*di*_	R&D output of downstream pharmaceutical enterprises.
*r*	The cost parameters of R&D in pharmaceutical enterprises.
*ν*_1_	The horizontal spillover effects of each unit of R&D activities carried out by upstream pharmaceutical enterprises on upstream competitors in the same industry.
*ν*_2_	The horizontal spillover effects of each unit of R&D activities carried out by downstream pharmaceutical enterprises on downstream competitors in the same industry.
*β*_1_	The vertical spillover effects of each unit of R&D activities carried out by upstream pharmaceutical enterprises on downstream enterprises.
*β*_2_	The vertical spillover effects of each unit of R&D activities carried out by downstream pharmaceutical enterprises on upstream enterprises.
*S*	The government subsidy rate based on the R&D investment of pharmaceutical enterprises.
*e*	The amount of government subsidies for each unit of innovative products produced by pharmaceutical enterprises.
*θ*	The amount of subsidy per unit given by the government to patients who purchase new drugs.
*η*	After the government included the new drug in medical insurance, the proportion of the amount paid by patients after medical insurance reimbursement to the original price.
*B*	Superscript, indicating non-government subsidy strategy.
*MS*	Superscript, indicating the pharmaceutical enterprise innovation input subsidy strategy.
*ME*	Superscript, indicating the pharmaceutical enterprise innovative product subsidy strategy.
*CI*	Superscript, indicating the patient price subsidy strategy.
*CR*	Superscript, indicating the patient medical insurance subsidy strategy.
*π*_*s*_	Profits of upstream pharmaceutical enterprises.
*π*_*d*_	Profits of downstream pharmaceutical enterprises.
*SW*	Social welfare.

### 3.2 Problem description and basic assumptions

Based on the two-stage duopoly model (the A-J model) proposed by D’Aspremont and Jacquemin, this study proposes a decision-making method of government subsidy strategies for new drug R&D, which comprehensively considers the double spillover effects. It needs to be clear that new drug R&D has high risks, and involve many links, which would promote the assignment of work and cooperation between pharmaceutical enterprises, so as to seek greater profits and reduce their own risks. There are obvious spillover effects in activities such as lead compound discovery, compound structure modification, raw and auxiliary material innovation, semi-finished product processing and other activities, especially in process improvement or technological innovation. From the perspective of the pharmaceutical production supply chain, this study regards leading compounds, raw and auxiliary materials and other suppliers as upstream pharmaceutical enterprises, accordingly regards pharmaceutical synthesis production enterprises as downstream pharmaceutical enterprises, and put the following hypotheses.

#### Assumption 1

In the pharmaceutical production supply chain, there are two identical upstream pharmaceutical enterprises and two identical downstream pharmaceutical enterprises. The upstream pharmaceutical enterprises produce intermediate product, and the downstream pharmaceutical enterprises process intermediate product into final product. A unit of intermediate product can only be converted into a unit of final product. The total output of intermediate product is *Q*_*s*_, the total output of final product is *Q*_*d*_, and the total output of upstream and downstream pharmaceutical enterprises is equal, that is *Q*_*s*_ = *Q*_*d*_ = *Q*. If there is no innovative R&D in the drug production process, the cost of each unit of intermediate product produced by the upstream pharmaceutical enterprises is $c_{1}$, and the intermediate product will be sold to the downstream pharmaceutical enterprises at the price *w* to process. The downstream pharmaceutical enterprises cost $c_{2}$ to convert this intermediate product into the final product, then sell the new drug to the patient at the price *p*. Assuming that the demand for new drug in the market meet a simple linear function, *p* = *a* − *bQ*, where *a*, *b* are constants greater than 0, *Q* = (*q*_*di*_ + *q*_*dj*_), where *i* = 1,2; *j* = 3 − *i*.

#### Assumption 2

Assuming that the main purpose of pharmaceutical enterprises for innovative R&D is to reduce production costs and obtain more profits. *x*_*si*_(*i* = 1,2) is the R&D output of upstream pharmaceutical enterprises, *x*_*di*_(*i* = 1,2) is the R&D output of downstream pharmaceutical enterprises. The R&D cost function of upstream pharmaceutical enterprises is $\frac{1}{2}{rx}_{si}^{2}$, and the R&D cost function of downstream pharmaceutical enterprises is $\frac{1}{2}{rx}_{di}^{2}$, the *r* is the cost parameter of the pharmaceutical enterprises’ innovative R&D, which is the output efficiency of the enterprises’ knowledge resources; the larger the *r*, the weaker the R&D capability of pharmaceutical enterprises will become; the smaller the *r*, the stronger the R&D capabilities of pharmaceutical enterprises will become.

#### Assumption 3

The upstream pharmaceutical enterprises can reduce cost by 1 unit for each unit of R&D, the horizontal spillover effects can enable upstream competitors to reduce the cost of *ν*_1_ unit, and the vertical spillover effects can enable downstream pharmaceutical enterprises to reduce the cost of *β*_1_ unit. Accordingly, downstream pharmaceutical enterprises can reduce cost by 1 unit for each unit of R&D, the horizontal spillover effects can enable downstream competitors to reduce the cost of *ν*_2_ unit, and the vertical spillover effects can enable upstream pharmaceutical enterprises to reduce the cost of *β*_2_ unit, where *ν*_1_, *ν*_2_, *β*_1_, *β*_2_ ∈ [0,1].

According to the above assumptions, the cost of per unit of intermediate product produced by the upstream pharmaceutical enterprises is as follows:

$$c_{si}=c_{1}-x_{si}-v_{1}x_{sj}-\beta_{2}\left(x_{d1}+x_{d2}\right),\;\text{where}\;i=1,2;j=3-i.$$ (1)

The cost for downstream pharmaceutical enterprises to convert a unit of intermediate product into final product is as follows:

$$c_{di}=w+c_{2}-x_{di}-v_{2}x_{dj}-\beta_{1}\left(x_{s1}+x_{s2}\right),\;\text{where}\;i=1,2;j=3-i.$$ (2)

To ensure that the production cost of innovative R&D by upstream and downstream pharmaceutical enterprises is positive in the presence of spillover effects in the industry, the $c_{1}$ and $c_{2}$ must meet the conditions which are as follows:

$$c_{1}>x_{si}+v_{1}x_{sj}+\beta_{2}\left(x_{d1}+x_{d2}\right).$$ (3)

$$c_{2}>x_{di}+v_{2}x_{dj}+\beta_{1}\left(x_{s1}+x_{s2}\right)-w$$ (4)

#### Assumption 4

In order to encourage pharmaceutical enterprises to conduct new drug R&D and encourage patients to choose new drug, the government will formulate subsidy strategies to encourage the enterprises or guide patients. According to the subsidy object, it can be divided into two categories: one is pharmaceutical enterprise subsidy strategy, the other is patient subsidy strategy. The pharmaceutical enterprise subsidy strategy can be divided into innovation input subsidy strategy and innovative product subsidy strategy according to the subsidy mode. The former is that government subsidize the innovation input of pharmaceutical enterprises according to a certain ratio *S*. The subsidy rate for upstream enterprises is *S*_1_, and for the downstream enterprises is *S*_s_. Although the subsidy rate for upstream and downstream enterprises is different, it won’t affect the results, so assuming that *S*_1_ = *S*_2_ = *S*. The latter is that the government subsidizes the products of enterprises according to a certain value *e*, where subsidizes *e*_1_ for per unit of intermediate product of the upstream pharmaceutical enterprises; and subsidizes *e*_2_ for per unit of final product of the downstream pharmaceutical enterprises. Accordingly, the different subsidy value also won’t affect the results, so assuming that *e*_1_ = *e*_2_ = *e*. The patient subsidy strategy can be divided into patient price subsidy strategy and patient medical insurance subsidy strategy according to the subsidy mode. The former is that the government pays for the patients’ purchase of new drug, subsidizes *θ* for each unit of new drug. The latter is that the government brings new drugs into medical insurance and sets the reimbursement ratio (1 − *η*), then patients can get subsidies for buying new drugs through medical insurance.

## 4 Basic model

The government subsidy strategies established in this paper have five main situations: non-government subsidy strategy, pharmaceutical enterprise innovation input subsidy strategy, pharmaceutical enterprise innovative product subsidy strategy, patient price subsidy strategy, and patient medical insurance subsidy strategy. The game model under various subsidy strategies contains four stages: the first stage is that the government decides the subsidy strategy and the value of subsidy; the second stage is that all enterprises make R&D investment decisions at the same time; the third stage is that the upstream enterprises determine the price *w* of intermediate product, the downstream enterprises determine the price *p* of the final product; and in the fourth stage, the upstream and downstream enterprises determine the output. Therefore, a four-stage dynamic game model with complete information is established, and a backward induction method is used to solve the sub-game perfect Nash equilibrium in various situations.

### 4.1 Non-government subsidy strategy (*B*)

When the government does not provide any R&D subsidies, the patient demand function is *p* = *a* − *bQ*, using $\pi_{si}^{B}$ and $\pi_{di}^{B}$ to represent the profit function of upstream and downstream pharmaceutical enterprises, which are as follows:

$$\pi_{si}^{B}=\left(w-c_{si}\right)q_{si}-\frac{1}{2}{rx}_{si}^{2}\;=\left[w-\left(c_{1}-x_{si}-v_{1}x_{sj}-\beta_{2}\left(x_{d1}+x_{d2}\right)\right)\right]q_{si}-\frac{1}{2}{rx}_{si}^{2},\;\text{where}\;i=1,2;j=3-i.$$ (5)

$$\pi_{di}^{B}=\left(p-c_{di}\right)q_{di}-\frac{1}{2}{rx}_{di}^{2}=\left[p-\left(w+c_{2}-x_{di}-v_{2}x_{dj}-\beta_{1}\left(x_{s1}+x_{s2}\right)\right)\right]q_{di}-\frac{1}{2}{rx}_{di}^{2},\;\text{where}\;i=1,2;j=3-i$$ (6)

The government is the representative of social interests, and through the formulation of policies to guide enterprises’ R&D activities, so as to maximize social welfare. Under the non-government subsidy strategy, social welfare is the consumer surplus plus the profits of all pharmaceutical enterprises, which is as follows:

$${SW}^{B}=\frac{1}{2}bQ^{2}+\sum_{i=1}^{2}\pi_{si}+\sum_{i=1}^{2}\pi_{di},\;\text{where}\;i=1,2$$ (7)

In the above models, the upstream pharmaceutical enterprises are the leaders who determine the price of intermediate product and the upstream innovation input level; the downstream enterprises are the followers, who determine the drug sales price and downstream innovation input level. Lemma 1 can be obtained according to the backward induction.

**Lemma 1** Under the non-government subsidy strategy, the optimal decision-making, enterprises’ profits and social welfare of the pharmaceutical production supply chain are respectively as follows:

$$x_{si}^{B}=\frac{12\left(a-c_{1}-c_{2}\right)\left(\beta_{1}-\nu_{2}+2\right)}{F},\;\text{where}\;i=1,2;j=3-i$$ (8)

$$x_{si}^{B}=\frac{2\left(a-c_{1}-c_{2}\right)\left(4\beta_{2}-7\nu_{1}+11\right)}{F},\;\text{where}\;i=1,2;j=3-i$$ (9)

$$q^{B}=\frac{\left(a-c_{1}-c_{2}\right)\left[2F+4\left(\nu_{2}+1+2\beta_{2}\right)\left(4\beta_{2}-7\nu_{1}+11\right)+12\left(4\beta_{1}+2\nu_{1}+2\right)\left(\beta_{1}-\nu_{2}+2\right)\right]}{9bF}$$ (10)

$$w^{B}=\frac{\left(2a-2c_{2}+4c_{1}\right)F+\left(a-c_{1}-c_{2}\right)\left[4\left(\nu_{2}+1-4\beta_{2}\right)\left(4\beta_{2}-7\nu_{1}+11\right)+24\left(2\beta_{1}-2\nu_{1}-2\right)\left(\beta_{1}-\nu_{2}+2\right)\right]}{6F}$$ (11)

$$P^{B}=\frac{\left(5a+4c_{2}+4c_{1}\right)F-2\left(a-c_{1}-c_{2}\right)\left[4\left(2\beta_{2}+\nu_{2}+1\right)\left(4\beta_{2}-7\nu_{1}+11\right)+24\left(2\beta_{1}+\nu_{1}+1\right)\left(\beta_{1}-\nu_{2}+2\right)\right]}{9F}$$ (12)

$$\pi_{si}^{B}=\frac{{\left(a-c_{1}-c_{2}\right)}^{2}\left[{\left(2F+4\left(\nu_{2}+2\beta_{2}+1\right)\left(4\beta_{2}-7\nu_{1}+11\right)+24\left(2\beta_{1}+\nu_{1}+1\right)\left(\beta_{1}-\nu_{2}+2\right)\right)}^{2}-3888br{\left(\beta_{1}-\nu_{2}+2\right)}^{2}\right]}{54bF^{2}}$$ (13)

$$\pi_{di}^{B}=\frac{{\left(a-c_{1}-c_{2}\right)}^{2}\left[{\left(2F+4\left(\nu_{2}+2\beta_{2}+1\right)\left(4\beta_{2}-7\nu_{1}+11\right)+24\left(2\beta_{1}+\nu_{1}+1\right)\left(\beta_{1}-\nu_{2}+2\right)\right)}^{2}-162br{\left(4\beta_{2}-7\nu_{1}+11\right)}^{2}\right]}{81bF^{2}}$$ (14)

$${SW}^{B}=\frac{{10\left(a-c_{1}-c_{2}\right)}^{2}\left[{\left[2F+4\left(\nu_{2}+2\beta_{2}+1\right)\left(4\beta_{2}-7\nu_{1}+11\right)+24\left(2\beta_{1}+\nu_{1}+1\right)\left(\beta_{1}-\nu_{2}+2\right)\right]}^{2}-81br\left[144{\left(\beta_{1}-\nu_{2}+2\right)}^{2}+4{\left(4\beta_{2}-7\nu_{1}+11\right)}^{2}\right]\right]}{{162bF}^{2}}$$ (15)

Where,

$$F=-46-8\nu_{1}+14\nu_{1}^{2}-12\nu_{2}+12\nu_{2}^{2}-60\beta_{1}-52\beta_{2}+20\beta_{2}\nu_{1}+12\beta_{1}\nu_{2}-24\beta_{1}^{2}-16\beta_{2}^{2}+81rb.$$ (16)

The certification process is shown in the S1 Appendix.

**Corollary 1** Under the non-government subsidy strategy, the impact of horizontal spillover effects and vertical spillover effects among pharmaceutical enterprises in the industry on the optimal decision-making of the pharmaceutical supply chain, enterprises’ profits and social welfare are respectively as follows:

(i)
$\frac{\partial x_{si}^{B}}{\partial \nu_{1}}<0,\frac{\partial x_{si}^{B}}{\partial v_{2}}<0;\frac{\partial x_{si}^{B}}{\partial \beta_{1}}>0,\frac{\partial x_{si}^{B}}{\partial \beta_{2}}>0;\;\text{where}\;i=1,2$

(ii)
$\frac{\partial x_{di}^{B}}{\partial \nu_{1}}<0,\frac{\partial x_{di}^{B}}{\partial v_{2}}<0;\frac{\partial x_{di}^{B}}{\partial \beta_{1}}>0,\frac{\partial x_{di}^{B}}{\partial \beta_{2}}>0;\;\text{where}\;i=1,2$

(iii)
$\frac{\partial q^{B}}{\partial \nu_{1}}>0,\frac{\partial q^{B}}{\partial v_{2}}>0;\frac{\partial q^{B}}{\partial \beta_{1}}>0,\frac{\partial q^{B}}{\partial \beta_{2}}>0;\;\text{where}\;i=1,2$

(iv)
$\frac{\partial w^{B}}{\partial v_{1}}<0,\frac{\partial w^{B}}{\partial \nu_{2}}<0;\frac{\partial w^{B}}{\partial \beta_{1}}>0,\frac{\partial w^{B}}{\partial \beta_{2}}>0;\;\text{where}\;i=1,2$

(v)
$\frac{\partial p^{B}}{\partial \nu_{1}}<0,\frac{\partial p^{B}}{\partial \nu_{2}}<0;\frac{\partial p^{B}}{\partial \beta_{1}}<0\frac{\partial p^{B}}{\partial \beta_{2}}<0;\;\text{where}\;i=1,2$

(vi)
$\frac{\partial \pi_{si}^{B}}{\partial v_{1}}>0,\frac{\partial \pi_{si}^{B}}{\partial v_{2}}>0;\frac{\partial \pi_{si}^{B}}{\partial \beta_{1}}>0\frac{\partial \pi_{si}^{B}}{\partial \beta_{2}}>0;\;\text{where}\;i=1,2$

(vii)
$\frac{\partial \pi_{si}^{B}}{\partial v_{1}}>0,\frac{\partial \pi_{si}^{B}}{\partial \nu_{2}}>0;\frac{\partial \pi_{di}^{B}}{\partial \beta_{1}}>0,\frac{\partial \pi_{si}^{B}}{\partial \beta_{2}}>0;\;\text{where}\;i=1,2$

(viii)
$\frac{\partial {SW}^{B}}{\partial \nu_{1}}>0,\frac{\partial {SW}^{B}}{\partial v_{2}}>0;\frac{\partial {SW}^{B}}{\partial \beta_{1}}>0,\frac{\partial {SW}^{B}}{\partial \beta_{2}}>0;\;\text{where}\;i=1,2$

It can be seen from Corollary 1 (i) and (ii) that under the non-government subsidy strategy, as the horizontal spillover rate *ν* increases, the R&D investment of upstream and downstream pharmaceutical enterprises decrease; as the vertical spillover rate *β* increases, the R&D investment of upstream and downstream pharmaceutical enterprises increase. With the increase of horizontal spillover rate, enterprises can innovate with the help of peer technology spillovers, which can reduce R&D barriers, and R&D investment is reduced compared with completely independent R&D; However, the benefits brought by the increase of vertical spillover rate are far less than the disadvantages caused by the weakening of intellectual property protection, only by increasing R&D investment can upstream and downstream pharmaceutical enterprises improve their core competitiveness.

From Corollary 1 (iii)-(v), it can be seen that under the non-government subsidy strategy, the output *q* increases with the increase of the horizontal spillover rate *ν* and the vertical spillover rate *β*; the price of intermediate product *w* decreases with the increase of horizontal spillover rate *ν*, but increases with the increase of vertical spillover rate *β*; the new drug price *p* decreases with the increase of the horizontal spillover rate *ν* and vertical spillover rate *β*. This is mainly because the increase of the horizontal spillover rate reduces the cost of pharmaceutical enterprises, so the price of intermediate product and new drug have been lowered; and the increase of the vertical spillover rate can enable upstream enterprises to occupy a dominant position and improve their own products by acquiring some downstream enterprises’ technologies. As a result, the prices of intermediate product have been raised, while downstream pharmaceutical enterprises have lowered the prices of new drug due to the reduction of their competitive advantages. Then, under the effect of the downward adjustment of new drug price, the output *q* will increase through the linear demand function of the product.

From Corollary 1 (vi) and (vii), it can be seen that with the increase of the spillover effects *ν*, *β*, the production cost of pharmaceutical enterprises will decrease. Even if the price of new drug decreases, the profits of upstream and downstream enterprises will increase under the influence of "small profits but quick turnover". Finally, it can be seen from (viii) that social welfare will increase with the increase of the spillover effects *ν*, *β*, the increase of spillover effects increases consumer surplus and increases corporate profits accordingly, which will lead to an increase in social welfare.

Based on the above analyses, the following proposition can be obtained as follows:

**Proposition 1** Under the non-government subsidy strategy, the optimal R&D investment decision, price decision and optimal profit of upstream and downstream pharmaceutical enterprises and social welfare are all related to *c*_1_, *c*_2_, *ν*, *β*. The enterprises’ R&D investment and the price of intermediate product decrease with the increase of the horizontal spillover rate *ν* and the vertical spillover rate *β*; the price of new drug decreases with the increase of *ν* and *β*; while the new drug output, profits of enterprises, and social welfare will all increase with the increase of *ν* and *β*.

### 4.2 Pharmaceutical enterprise subsidy strategy (*M*)

In order to encourage new drug R&D, the government will provide subsidies for pharmaceutical enterprises. There are two main forms: one is innovation input subsidy strategy, the other is innovative product subsidy strategy. For enterprises, the purpose of obtaining subsidies is to obtain greater profits, while for the government, the purpose is to encourage enterprises’ innovation and increase social welfare.

#### 4.2.1 Innovation input subsidy strategy (*MS*)

Under the pharmaceutical enterprise innovation input subsidy strategy, the government will subsidize pharmaceutical enterprises at a certain subsidy rate *s* (*s* ∈ [0,1]) according to the innovation input *x*, using *sx* to represent the amount of subsidies received by the enterprises. Under this situation, using $\pi_{si}^{MS}$ and $\pi_{di}^{MS}$ to represent the profit function of the upstream and downstream enterprises, which are as follows:

$$\pi_{si}^{MS}=\left(w-c_{si}\right)q_{si}-\frac{1}{2}{rx}_{si}^{2}+sx_{si}=\left[w-\left(c_{1}-x_{si}-v_{1}x_{sj}-\beta_{2}\left(x_{d1}+x_{d2}\right)\right)\right]q_{si}-\frac{1}{2}{rx}_{si}^{2}+s_{si}x_{si},\;\text{where}\;i=1,2;j=3-i$$ (17)

$$\pi_{di}^{MS}=\left(p-c_{di}\right)q_{di}-\frac{1}{2}{rx}_{di}^{2}+sx_{di}=\left[p-\left(w+c_{2}-x_{di}-v_{2}x_{dj}-\beta_{1}\left(x_{s1}+x_{s2}\right)\right)\right]q_{di}-\frac{1}{2}{rx}_{di}^{2}+s_{di}x_{di},\;\text{where}\;i=1,2;j=3-i$$ (18)

Under the pharmaceutical enterprise innovation input subsidy strategy, social welfare is expressed as consumer surplus plus the profits of all pharmaceutical enterprises, and then subtract the government’s subsidies expenditures on innovation input of enterprises, which is as follows:

$${SW}^{MS}=\frac{1}{2}bQ^{2}+\sum_{i=1}^{2}\pi_{si}+\sum_{i=1}^{2}\pi_{di}-s\left(\sum_{i=1}^{2}x_{si}+\sum_{i=1}^{2}x_{di}\right),\;\text{where}\;i=1,2$$ (19)

In the above models, the government is the leader who determines the subsidy rate of innovation input of pharmaceutical enterprises; the upstream and downstream enterprises are followers, who determine the level of innovation input and product pricing. Lemma 2 can be obtained according to the backward induction.

**Lemma 2** Under the pharmaceutical enterprise innovation input subsidy strategy, the optimal decision-making, enterprises’ profits and social welfare of the pharmaceutical production supply chain are respectively as follows:

$$x_{si}^{MS}=\frac{12\left(a-c_{1}-c_{2}\right)\left(\beta_{1}-\nu_{2}+2\right)+81bs}{F},\;\text{where}\;i=1,2;j=3-i$$ (20)

$$x_{di}^{MS}=\frac{2\left(a-c_{1}-c_{2}\right)\left(4\beta_{2}-7\nu_{1}+11\right)+81bs}{F},\;\text{where}\;i=1,2;j=3-i$$ (21)

$$q^{MS}=\frac{\left(a-c_{1}-c_{2}\right)\left[2F+\left(\nu_{2}+1+2\beta_{2}\right)\left[4\left(4\beta_{2}-7\nu_{1}+11\right)+\frac{162bs}{a-c_{1}-c_{2}}\right]+\left(2\beta_{1}+1\nu_{1}+1\right)\left[24\left(\beta_{1}-\nu_{2}+2\right)+\frac{162bs}{a-c_{1}-c_{2}}\right]\right]}{9bF}$$ (22)

$$w^{MS}=\frac{\left(2a-2c_{2}+4c_{1}\right)F+\left(a-c_{1}-c_{2}\right)\left[\left(\nu_{2}+1-4\beta_{2}\right)\left[4\left(4\beta_{2}-7\nu_{1}+11\right)+\frac{162bs}{a-c_{1}-c_{2}}\right]+\left(2\beta_{1}-2\nu_{1}-2\right)\left[24\left(\beta_{1}-\nu_{2}+2\right)+\frac{162bs}{a-c_{1}-c_{2}}\right]\right]}{6F}$$ (23)

$$P^{MS}=\frac{\left(5a+4c_{2}+4c_{1}\right)F-2\left(a-c_{1}-c_{2}\right)\left[\left(2\beta_{2}+\nu_{2}+1\right)\left[4\left(4\beta_{2}-7\nu_{1}+11\right)+\frac{162bs}{a-c_{1}-c_{2}}\right]+\left(2\beta_{1}+\nu_{1}+1\right)\left[24\left(\beta_{1}-\nu_{2}+2\right)+\frac{162bs}{a-c_{1}-c_{2}}\right]\right]}{9F}$$ (24)

$$\begin{array}{l} \pi_{si}^{MS}=\frac{{\left[\left(a-c_{1}-c_{2}\right)\left[2F+4\left(\nu_{2}+2\beta_{2}+1\right)\left(4\beta_{2}-7\nu_{1}+11\right)+24\left(2\beta_{1}+\nu_{1}+1\right)\left(\beta_{1}-\nu_{2}+2\right)\right]+162bs\left(\nu_{1}+\nu_{2}+2\beta_{1}+2\beta_{2}+2\right)\right]}^{2}}{54bF^{2}}\\ -\frac{1}{2}r{\left[\frac{12\left(a-c_{1}-c_{2}\right)\left(\beta_{1}-\nu_{2}+2\right)+81bs}{F}\right]}^{2}+s\left[\frac{12\left(a-c_{1}-c_{2}\right)\left(\beta_{1}-\nu_{2}+2\right)+81bs}{F}\right] \end{array}$$ (25)

$$\begin{array}{l} \pi_{di}^{MS}=\frac{{\left[\left(a-c_{1}-c_{2}\right)\left[2F+4\left(\nu_{2}+2\beta_{2}+1\right)\left(4\beta_{2}-7\nu_{1}+11\right)+24\left(2\beta_{1}+\nu_{1}+1\right)\left(\beta_{1}-\nu_{2}+2\right)\right]+162bs\left(\nu_{1}+\nu_{2}+2\beta_{1}+2\beta_{2}+2\right)\right]}^{2}}{81bF^{2}}\\ -\frac{1}{2}r{\left[\frac{2\left(a-c_{1}-c_{2}\right)\left(4\beta_{2}-7\nu_{1}+11\right)+81bs}{F}\right]}^{2}+s\left[\frac{2\left(a-c_{1}-c_{2}\right)\left(4\beta_{2}-7\nu_{1}+11\right)+81bs}{F}\right] \end{array}$$ (26)

$$\begin{array}{l} {SW}^{MS}=\frac{14{\left[\left(a-c_{1}-c_{2}\right)\left[2F+4\left(\nu_{2}+2\beta_{2}+1\right)\left(4\beta_{2}-7\nu_{1}+11\right)+24\left(2\beta_{1}+\nu_{1}+1\right)\left(\beta_{1}-\nu_{2}+2\right)\right]+162bs\left(\nu_{1}+\nu_{2}+2\beta_{1}+2\beta_{2}+2\right)\right]}^{2}}{162bF^{2}}\\ -\frac{162br\left[{\left[12\left(a-c_{1}-c_{2}\right)\left(\beta_{1}-\nu_{2}+2\right)+81bs\right]}^{2}+{\left[2\left(a-c_{1}-c_{2}\right)\left(4\beta_{2}-7\nu_{1}+11\right)+81bs\right]}^{2}\right]}{162bF^{2}} \end{array}$$ (27)

The proof process is similar to Lemma 1, it will not be repeated here.

**Corollary 2** Under the pharmaceutical enterprise innovation input subsidy strategy, the impact of subsidies on these enterprises’ decision-making and social welfare are respectively as follows:

(i)
$\frac{\partial x_{si}^{MS}}{\partial S}>0,\frac{\partial x_{di}^{MS}}{\partial S}>0,\;\text{where}\;i=1,2$

(ii)
$\frac{\partial q^{MS}}{\partial S}>0$

(iii)
$\frac{\partial w^{MS}}{\partial S}<0,\frac{\partial p^{MS}}{\partial S}<0$

(iv)
$\frac{\partial \pi_{si}^{MS}}{\partial S}>0,\frac{\partial \pi_{di}^{MS}}{\partial S}>0,\;\text{where}\;i=1,2$

(v)
$\frac{\partial {SW}^{MS}}{\partial S}>0$

From Corollary 2 (i), it can be seen that under the pharmaceutical enterprise innovation input subsidy strategy, the government subsidies have a positive impact on the innovation input of upstream and downstream enterprises, indicating that the greater the government subsidies, the more willing the enterprises increase the level of new drug R&D investment. From Corollary 2 (ii) and (iii), it is obviously found that under the pharmaceutical enterprise innovation input subsidy strategy, government subsidies have a positive impact on patient demand, and have a negative impact on the price setting of upstream and downstream enterprises. This is because with the increase of government subsidies, enterprises will appropriately lower prices while ensuring self-profits, thereby increase the patients’ demand. It can be seen from Corollary 2 (iv) and (v) that government subsidies have a positive impact on the profits of all enterprises and social welfare. It is obvious that the government’s subsidies for the innovation input of pharmaceutical enterprises can have a positive impact on enterprises and society, so the government can promote new drug R&D and improve social welfare through this subsidy strategy.

**Corollary 3** According to the maximization of social welfare, take the derivative of *SW*^*MS*^ with respect to *S*, and set $\frac{\partial {SW}^{MS}}{\partial S}=0$, then it is obtained as follows:

$$S=\frac{H-28\left(a-c_{1}-c_{2}\right)\left(2F+G\right)\left(\nu_{1}+\nu_{2}+2\beta_{1}+2\beta_{2}+2\right)}{162bK}$$ (28)

where,

$$G=4\left(\nu_{2}+2\beta_{2}+1\right)\left(4\beta_{2}-7\nu_{1}+11\right)+24\left(2\beta_{1}+\nu_{1}+1\right)\left(\beta_{1}-\nu_{2}+2\right)$$ (29)

$$H=162br\left(a-c_{1}-c_{2}\right)\left[12\left(\beta_{1}-\nu_{2}+2\right)+2\left(4\beta_{2}-7\nu_{1}+11\right)\right]$$ (30)

$$K=28{\left(\nu_{1}+\nu_{2}+2\beta_{1}+2\beta_{2}+2\right)}^{2}-162br$$ (31)

Then analyzing the impact of the horizontal spillover effects and the vertical spillover effects on the subsidy rate of innovation input of enterprises: if 0 < *ν*_1_, *ν*_2_ < *L*_1_, then $\frac{\partial S}{\partial \nu_{1}}>0,\frac{\partial S}{\partial \nu_{2}}>0$; otherwise, the result is opposite; if 0 < *β*_1_, *β*_2_ < *L*_2_, then $\frac{\partial S}{\partial \beta_{1}}>0,\frac{\partial S}{\partial \beta_{2}}>0$; otherwise, the result is opposite.

Where, $L_{1}=\frac{4.5{\left(1+\beta_{1}+2\beta_{2}\right)}^{2}}{9br-4.5\left(a-c_{1}-c_{2}\right){\left(1+\beta_{1}+2\beta_{2}\right)}^{2}}$, $L_{2}=\frac{4.5{\left(1+4\nu_{1}+2\nu_{2}\right)}^{2}}{9br-4\left(a-c_{1}-c_{2}\right){\left(1+4\nu_{1}+2\nu_{2}\right)}^{2}}$.

From Corollary 3, it can be seen that under the pharmaceutical enterprise innovation input subsidy strategy, the innovation input subsidy rate *S* is related to the spillover effects. If the horizontal spillover rate *ν* is small, then *S* increases as the horizontal spillover rate increases; if the horizontal spillover rate *ν* is large, then *S* decreases as the horizontal overflow rate increases. The vertical spillover rate *β* has the same effect on *S*. This is because when the spillover effects are small, enterprises can use peer spillovers to enhance the R&D process and increase R&D investment, so the government subsidy rate increases accordingly; when the spillover effect is large, the "free rider" effect dampens the enterprises’ enthusiasm of R&D investment, as they rely too much on their peers’ spillovers, their investment has decreased, and the government subsidy rate also decrease accordingly.

**Proposition 2** Comparing the non-government subsidy strategy and the pharmaceutical enterprise innovation input subsidy strategy, which can be obtained as follows:

If 0 < *ν*_1_ = *ν*_2_ < *L*_3_ or 0 < *β*_1_ = *β*_2_ < *L*_4_, then $\pi_{si}^{MS}>\pi_{si}^{B}$, $\pi_{di}^{MS}>\pi_{di}^{B}$, ${SW}_{di}^{MS}>{SW}_{di}^{B}$.

Where, $L_{3}=\frac{40.5r\left(a-c_{1}-c_{2}\right){\left(1+\beta_{1}+2\beta_{2}\right)}^{2}}{{\left[9br-4\left(1+\beta_{1}+2\beta_{2}\right)\right]}^{2}}$,$L_{4}=\frac{40.5r\left(a-c_{1}-c_{2}\right){\left(1+4v_{1}+2v_{2}\right)}^{2}}{{\left[9br-4\left(1+4v_{1}+2v_{2}\right)\right]}^{2}}$.

It can be seen from Proposition 2 that when the spillover rate is within a certain range, the pharmaceutical enterprise innovation input subsidy strategy will have an incentive effect on new drug R&D, which will help to increase the enterprises’ profits and social welfare. When it exceeds the scope, it will have a negative impact. Therefore, it is necessary for the government to examine the impact of spillover effects on decision-making when choosing pharmaceutical enterprise innovation input subsidy strategy.

#### 4.2.2 Innovative product subsidy strategy (*ME*)

Under the pharmaceutical enterprise innovative product subsidy strategy, the government will subsidize the pharmaceutical enterprises according to new drug sales volume *q*. From the description of Assumption 4, using *e* to represent the value of government subsidies for unit product to enterprises. Accordingly, using $\pi_{si}^{ME}$ and $\pi_{di}^{ME}$ to represent the profit function of the upstream and the downstream pharmaceutical enterprises, which are respectively as follows:

$$\pi_{si}^{ME}=\left(w-c_{si}+e_{}\right)q_{si}-\frac{1}{2}{rx}_{si}^{2}=\left[w-\left(c_{1}-x_{si}-v_{1}x_{sj}-\beta_{2}\left(x_{d1}+x_{d2}\right)\right)+e_{}\right]q_{si}-\frac{1}{2}{rx}_{si}^{2},\;\text{where}\;i=1,2;j=3-i$$ (32)

$$\pi_{di}^{ME}=\left(p-c_{di}+e_{}\right)q_{di}-\frac{1}{2}{rx}_{di}^{2}=\left[p-\left(w+c_{2}-x_{di}-v_{2}x_{dj}-\beta_{1}\left(x_{s1}+x_{s2}\right)\right)+e_{}\right]q_{di}-\frac{1}{2}{rx}_{di}^{2},\;\text{where}\;i=1,2;j=3-i$$ (33)

Under the pharmaceutical enterprise innovative product subsidy strategy, social welfare is expressed as the profits of all upstream and downstream enterprises, plus consumer surplus, and then subtract government subsidies for innovative products, which is as follows:

$${SW}^{ME}=\frac{1}{2}bQ^{2}+\sum_{i=1}^{2}\pi_{si}+\sum_{i=1}^{2}\pi_{di}-2eQ,\;\text{where}\;i=1,2$$ (34)

In the above models, the government is the leader who determines the amount of subsidies for innovative products of enterprises; the upstream and downstream enterprises are followers, who determine the level of innovation input and product pricing. Lemma 3 can be obtained according to the backward induction.

**Lemma 3** Under the pharmaceutical enterprise innovative product subsidy strategy, the optimal decision-making of the pharmaceutical production supply chain, enterprises’ profits, and social welfare are respectively as follows:

$$x_{si}^{ME}=\frac{12\left(a-c_{1}-c_{2}+2e\right)\left(\beta_{1}-\nu_{2}+2\right)}{F},\;\text{where}\;i=1,2;j=3-i$$ (35)

$$x_{di}^{ME}=\frac{2\left(a-c_{1}-c_{2}+2e\right)\left(4\beta_{2}-7\nu_{1}+11\right)}{F},\;\text{where}\;i=1,2;j=3-i$$ (36)

$$q^{ME}=\frac{\left(a-c_{1}-c_{2}+2e\right)\left[2F+4\left(\nu_{2}+1+2\beta_{2}\right)\left(4\beta_{2}-7\nu_{1}+11\right)+24\left(2\beta_{1}+\nu_{1}+1\right)\left(\beta_{1}-\nu_{2}+2\right)\right]}{9bF}$$ (37)

$$w^{ME}=\frac{\left(2a-2c_{2}+4c_{1}-2e\right)F+\left(a-c_{1}-c_{2}+2e\right)\left[4\left(\nu_{2}+1-4\beta_{2}\right)\left(4\beta_{2}-7\nu_{1}+11\right)+24\left(2\beta_{1}-2\nu_{1}-2\right)\left(\beta_{1}-\nu_{2}+2\right)\right]}{6F}$$ (38)

$$P^{ME}=\frac{\left(5a+4c_{2}+4c_{1}-8e\right)F-2\left(a-c_{1}-c_{2}+2e\right)\left[4\left(2\beta_{2}+\nu_{2}+1\right)\left(4\beta_{2}-7\nu_{1}+11\right)+24\left(2\beta_{1}+\nu_{1}+1\right)\left(\beta_{1}-\nu_{2}+2\right)\right]}{9F}$$ (39)

$$\pi_{si}^{ME}=\frac{{\left[\left(a-c_{1}-c_{2}+2e\right)\left[2F+4\left(\nu_{2}+2\beta_{2}+1\right)\left(4\beta_{2}-7\nu_{1}+11\right)+24\left(2\beta_{1}+\nu_{1}+1\right)\left(\beta_{1}-\nu_{2}+2\right)\right]\right]}^{2}}{54bF^{2}}-\frac{1}{2}r{\left[\frac{12\left(a-c_{1}-c_{2}+2e\right)\left(\beta_{1}-\nu_{2}+2\right)}{F}\right]}^{2}$$ (40)

$$\pi_{di}^{ME}=\frac{{\left[\left(a-c_{1}-c_{2}+2e\right)\left[2F+4\left(\nu_{2}+2\beta_{2}+1\right)\left(4\beta_{2}-7\nu_{1}+11\right)+24\left(2\beta_{1}+\nu_{1}+1\right)\left(\beta_{1}-\nu_{2}+2\right)\right]\right]}^{2}}{81bF^{2}}-\frac{1}{2}r{\left[\frac{2\left(a-c_{1}-c_{2}+2e\right)\left(4\beta_{2}-7\nu_{1}+11\right)}{F}\right]}^{2}$$ (41)

$$\begin{array}{l} {SW}^{ME}=\frac{14{\left[\left(a-c_{1}-c_{2}+2e\right)\left[2F+4\left(\nu_{2}+2\beta_{2}+1\right)\left(4\beta_{2}-7\nu_{1}+11\right)+24\left(2\beta_{1}+\nu_{1}+1\right)\left(\beta_{1}-\nu_{2}+2\right)\right]\right]}^{2}}{162bF^{2}}\\ -\frac{4e{\left[\left(a-c_{1}-c_{2}+2e\right)\left[2F+4\left(\nu_{2}+2\beta_{2}+1\right)\left(4\beta_{2}-7\nu_{1}+11\right)+24\left(2\beta_{1}+\nu_{1}+1\right)\left(\beta_{1}-\nu_{2}+2\right)\right]\right]}^{}}{9bF}\\ -\frac{162br\left[{\left[12\left(a-c_{1}-c_{2}+2e\right)\left(\beta_{1}-\nu_{2}+2\right)\right]}^{2}+{\left[2\left(a-c_{1}-c_{2}+2e\right)\left(4\beta_{2}-7\nu_{1}+11\right)\right]}^{2}\right]}{162bF^{2}} \end{array}$$ (42)

The proof process is similar to Lemma 1, it will be not repeated here.

**Corollary 4** Under the pharmaceutical enterprise innovative product subsidy strategy, the impact of subsidies on pharmaceutical enterprises’ decision-making and social welfare are respectively as follows:

(i)
$\frac{\partial x_{si}^{ME}}{\partial e}>0,\frac{\partial x_{di}^{ME}}{\partial e}>0,\;\text{where}\;i=1,2$

(ii)
$\frac{\partial q^{ME}}{\partial e}>0$

(iii)
$\frac{\partial w^{ME}}{\partial e}<0,\frac{\partial p^{ME}}{\partial e}<0$

(iv)
$\frac{\partial \pi_{si}^{ME}}{\partial e}>0,\frac{\partial \pi_{di}^{ME}}{\partial e}>0,\;\text{where}\;i=1,2$

(v)
$\frac{\partial {SW}^{ME}}{\partial e}>0$

Corollary 4 (i) shows that under the pharmaceutical enterprise innovative product subsidy strategy, as the government subsidies increase, the upstream and downstream enterprises will increase their investment in new drug R&D. It can be seen from (ii) and (iii) that government subsidies for innovative products will have a positive effect on the demand for new drug, which is mainly because the subsidies will make these enterprises lower the prices of intermediate product and new drug, leading to an increase in demand. From (iv) and (v), it can be seen that government subsidies will increase all enterprises’ profits and social welfare.

**Corollary 5** According to the maximization of social welfare, take the derivation of *SW*^*ME*^ with respect to *e*, and set $\frac{\partial {SW}^{ME}}{\partial e}=0$, then it is obtained as follows:

$$e=\frac{\left(a-c_{1}-c_{2}\right)\left[\left(5F+7G\right)\left(2F+G\right)-81br\left[144{\left(\beta_{1}-\nu_{2}+2\right)}^{2}+4{\left(4\beta_{2}-7\nu_{1}+11\right)}^{2}\right]\right]}{\left(8F+14G\right)\left(2F+G\right)+162br\left[144{\left(\beta_{1}-\nu_{2}+2\right)}^{2}+4{\left(4\beta_{2}-7\nu_{1}+11\right)}^{2}\right]}$$ (43)

Then analyzing the impact of the horizontal spillover effects and vertical spillover effects on the value of subsidy *e* per unit product of pharmaceutical enterprises, which is obtained as follows:

$$\frac{\partial e}{\partial \nu_{1}}>0,\frac{\partial e}{\partial v_{2}}>0;\frac{\partial e}{\partial \beta_{1}}<0,\frac{\partial e}{\partial \beta_{2}}<0$$

From Corollary 5, it can be seen that under the pharmaceutical enterprise innovative product subsidy strategy, the government’s subsidy value *e* of per unit product increases with the increase of the horizontal spillover rate *ν*, and decreases with the increase of the vertical spillover rate *β*. This is because the increase of horizontal spillover effects accelerates the exchange of product R&D technologies in the industry, and the output efficiency of generic products has increased, so the government subsidies have been increased accordingly. The increase of the vertical spillover rate increase the communication between upstream and downstream enterprises, the difference between upstream and downstream products will be reduced, so the government subsidies will be reduced accordingly.

**Proposition 3** Comparing the non-government subsidy strategy and the pharmaceutical enterprise innovative product subsidy strategy, which can be obtained as follows:

$$\pi_{si}^{ME}>\pi_{si}^{B},\pi_{di}^{ME}>\pi_{di}^{B},{SW}_{di}^{ME}>{SW}_{di}^{B}.$$

From Proposition 3, it can be seen that compared with the non-government subsidy strategy, the pharmaceutical enterprise innovative product subsidy strategy will stimulate the pharmaceutical enterprises’ new drug R&D, which will help to increase the enterprises’ profits and social welfare. Therefore, the government can promote the development of the pharmaceutical industry through this strategy.

### 4.3 Patient subsidy strategy (*C*)

In order to encourage pharmaceutical enterprises to carry out new drug R&D, the government will also give patients preferential treatment through subsidies. It will encourage patients to purchase new drugs and stimulate pharmaceutical enterprises to develop new drug R&D and increase social welfare. There are two main types of subsidies for patients: one is the patient price subsidy strategy, the essence of which is that the government pays for patients to purchase new drugs and guides patients to purchase new drugs by reducing costs; the other is the patient medical insurance subsidy strategy, through the way of medical insurance, the patients will be subsidized according to the proportion of diseases, and the patients will buy drugs first and then be reimbursed. Next, analyzing the impact of these two patient subsidy strategies on pharmaceutical enterprises’ new drug R&D.

#### 4.3.1 Patient price subsidy strategy (*CI*)

Under the patient price subsidy strategy, government subsidies directly affect the retail price, the price of the new drug purchased by patients is *p* − *θ*, where *θ* is the price subsidy per product, and the patient price demand function is *p* = *a* − *bQ* + *θ*. Using $\pi_{si}^{CI}$ and $\pi_{di}^{CI}$ to represent the profit function of the upstream and downstream pharmaceutical enterprises, which are respectively as follows:

$$\pi_{si}^{CI}=\left(w-c_{si}\right)q_{si}-\frac{1}{2}{rx}_{si}^{2}=\left[w-\left(c_{1}-x_{si}-v_{1}x_{sj}-\beta_{2}\left(x_{d1}+x_{d2}\right)\right)\right]q_{si}-\frac{1}{2}{rx}_{si}^{2},\;\text{where}\;i=1,2;j=3-i$$ (44)

$$\pi_{di}^{CI}=\left(p-c_{di}\right)q_{di}-\frac{1}{2}{rx}_{di}^{2}=\left[p-\left(w+c_{2}-x_{di}-v_{2}x_{dj}-\beta_{1}\left(x_{s1}+x_{s2}\right)\right)\right]q_{di}-\frac{1}{2}{rx}_{di}^{2},\;\text{where}\;i=1,2;j=3-i$$ (45)

Under the patient price subsidy strategy, social welfare is expressed as the profits of all enterprises plus consumer surplus, and then subtract government subsidies, which is as follows:

$${SW}^{CI}=\frac{1}{2}bQ^{2}+\sum_{i=1}^{2}\pi_{si}+\sum_{i=1}^{2}\pi_{di}-\theta Q,\;\text{where}\;i=1,2$$ (46)

In the above models, the government is the leader who determines the amount of patient price subsidies; the upstream and downstream pharmaceutical enterprises are followers, who determine the level of innovation input and product pricing. Lemma 4 can be obtained according to the backward induction.

**Lemma 4** Under the patient price subsidy strategy, the optimal decision-making, enterprises’ profits, and social welfare of the pharmaceutical production supply chain are respectively as follows:

$$x_{si}^{CI}=\frac{12\left(a-c_{1}-c_{2}+\theta \right)\left(\beta_{1}-\nu_{2}+2\right)}{F},\;\text{where}\;i=1,2;j=3-i$$ (47)

$$x_{di}^{CI}=\frac{2\left(a-c_{1}-c_{2}+\theta \right)\left(4\beta_{2}-7\nu_{1}+11\right)}{F},\;\text{where}\;i=1,2;j=3-i$$ (48)

$$q^{CI}=\frac{\left(a-c_{1}-c_{2}+\theta \right)\left[2F+4\left(\nu_{2}+1+2\beta_{2}\right)\left(4\beta_{2}-7\nu_{1}+11\right)+24\left(2\beta_{1}+\nu_{1}+1\right)\left(\beta_{1}-\nu_{2}+2\right)\right]}{9bF}$$ (49)

$$w^{CI}=\frac{\left(2a-2c_{2}+4c_{1}+2\theta \right)F+\left(a-c_{1}-c_{2}+\theta \right)\left[4\left(\nu_{2}+1-4\beta_{2}\right)\left(4\beta_{2}-7\nu_{1}+11\right)+24\left(2\beta_{1}-2\nu_{1}-2\right)\left(\beta_{1}-\nu_{2}+2\right)\right]}{6F}$$ (50)

$$P^{CI}=\frac{\left(5a+4c_{2}+4c_{1}+5\theta \right)F-2\left(a-c_{1}-c_{2}+\theta \right)\left[4\left(2\beta_{2}+\nu_{2}+1\right)\left(4\beta_{2}-7\nu_{1}+11\right)+24\left(2\beta_{1}+\nu_{1}+1\right)\left(\beta_{1}-\nu_{2}+2\right)\right]}{9F}$$ (51)

$$\pi_{si}^{CI}=\frac{{\left[\left(a-c_{1}-c_{2}+\theta \right)\left[2F+4\left(\nu_{2}+2\beta_{2}+1\right)\left(4\beta_{2}-7\nu_{1}+11\right)+24\left(2\beta_{1}+\nu_{1}+1\right)\left(\beta_{1}-\nu_{2}+2\right)\right]\right]}^{2}}{54bF^{2}}-\frac{1}{2}r{\left[\frac{12\left(a-c_{1}-c_{2}+\theta \right)\left(\beta_{1}-\nu_{2}+2\right)}{F}\right]}^{2}$$ (52)

$$\pi_{di}^{CI}=\frac{{\left[\left(a-c_{1}-c_{2}+\theta \right)\left[2F+4\left(\nu_{2}+2\beta_{2}+1\right)\left(4\beta_{2}-7\nu_{1}+11\right)+24\left(2\beta_{1}+\nu_{1}+1\right)\left(\beta_{1}-\nu_{2}+2\right)\right]\right]}^{2}}{81bF^{2}}-\frac{1}{2}r{\left[\frac{2\left(a-c_{1}-c_{2}+\theta \right)\left(4\beta_{2}-7\nu_{1}+11\right)}{F}\right]}^{2}$$ (53)

$$\begin{array}{l} {SW}^{CI}=\frac{14{\left[\left(a-c_{1}-c_{2}+\theta \right)\left[2F+4\left(\nu_{2}+2\beta_{2}+1\right)\left(4\beta_{2}-7\nu_{1}+11\right)+24\left(2\beta_{1}+\nu_{1}+1\right)\left(\beta_{1}-\nu_{2}+2\right)\right]\right]}^{2}}{162bF^{2}}\\ -\frac{2\theta {\left[\left(a-c_{1}-c_{2}+\theta \right)\left[2F+4\left(\nu_{2}+2\beta_{2}+1\right)\left(4\beta_{2}-7\nu_{1}+11\right)+24\left(2\beta_{1}+\nu_{1}+1\right)\left(\beta_{1}-\nu_{2}+2\right)\right]\right]}^{}}{9bF}\\ -\frac{162br\left[{\left[12\left(a-c_{1}-c_{2}+\theta \right)\left(\beta_{1}-\nu_{2}+2\right)\right]}^{2}+{\left[2\left(a-c_{1}-c_{2}+\theta \right)\left(4\beta_{2}-7\nu_{1}+11\right)\right]}^{2}\right]}{162bF^{2}} \end{array}$$ (54)

The proof process is similar to Lemma 1, it will not repeated here.

**Corollary 6** Under the patient price subsidy strategy, the impact of subsidies on pharmaceutical enterprises’ decision-making and social welfare are respectively as follows:

(i)
$\frac{\partial x_{si}^{CI}}{\partial \theta }>0,\frac{\partial x_{di}^{CI}}{\partial \theta }>0,\;\text{where}\;i=1,2$

(ii)
$\frac{\partial q^{CI}}{\partial \theta }>0$

(iii)
$\frac{\partial w^{CI}}{\partial \theta }>0,\frac{\partial p^{CI}}{\partial \theta }>0$

(iv)
$\frac{\partial \pi_{si}^{CI}}{\partial \theta }>0,\frac{\partial \pi_{di}^{CI}}{\partial \theta }>0,\;\text{where}\;i=1,2$

(v)
$\frac{\partial {SW}^{CI}}{\partial \theta }>0$

Corollary 6 (i) shows that under the patient price subsidy strategy, as government subsidies increase, all enterprises will increase their investment in new drug R&D. It can be seen from (ii) and (iii) that when the government’s price subsidies for patients increase, it will have a positive effect on the demand for new drug. Meanwhile, as the demand increases,enterprises will increase the price of intermediate product and new drug. It can be seen from (iv) and (v) that the government subsidy will increase enterprises’ profits and social welfare. In summary, obviously the patient price subsidy strategy will have an incentive effect on pharmaceutical enterprises’ new drug R&D, and can increase consumer surplus and social welfare. Thus, the government can use this strategy to improve the level of new drug R&D in the pharmaceutical industry.

**Corollary 7** According to the maximization of social welfare, take the derivative of *SW*^*CI*^ with respect to *θ*, and set $\frac{\partial SW^{CI}}{\partial \theta }=0$, then it is obtained as follows:

$$\theta =\frac{\left(a-c_{1}-c_{2}\right)\left[\left(10F+14G\right)\left(2F+G\right)-162br\left[144{\left(\beta_{1}-\nu_{2}+2\right)}^{2}+4{\left(4\beta_{2}-7\nu_{1}+11\right)}^{2}\right]\right]}{\left(8F+14G\right)\left(2F+G\right)+162br\left[144{\left(\beta_{1}-\nu_{2}+2\right)}^{2}+4{\left(4\beta_{2}-7\nu_{1}+11\right)}^{2}\right]}$$ (55)

Then analyzing the impact of the horizontal spillover effects and vertical spillover effects on the value of patient new drug price subsidy *θ*, which is obtained as follows:

$$\frac{\partial \theta }{\partial \nu_{1}}>0,\frac{\partial \theta }{\partial v_{2}}>0;\frac{\partial \theta }{\partial \beta_{1}}<0,\frac{\partial \theta }{\partial \beta_{2}}<0.$$

It can be seen from Corollary 7 that under the patient price subsidy strategy, the new drug price subsidy value *θ* increases with the increase of the horizontal spillover rate *ν*, and decreases with the increase of the vertical spillover rate *θ*. This is because the increase in horizontal spillover effects accelerate the exchange of product R&D technologies in the industry, and the quality of similar products has improved, leading to price increases. In order to benefit patients more, the government will increase subsidies. However, the increase in vertical spillover effects increase technological exchanges between industrial enterprises. As a result, the difference between upstream and downstream products is reduced, product prices are therefore reduced, and government subsidies to patients will be reduced.

**Proposition 4** Comparing the non-government subsidy strategy and the patient price subsidy strategy, which is obtained as follows:

$$x_{si}^{CI}>x_{si}^{B},x_{di}^{CI}>x_{di}^{B},\pi_{si}^{CI}>\pi_{si}^{B},\pi_{di}^{CI}>\pi_{di}^{B},{SW}_{di}^{CI}>{SW}_{di}^{B}.$$

From Proposition 4, it can be seen that compared with the non-government subsidy strategy, the patient price subsidy strategy has a positive incentive effect on the new drug R&D, which will help to increase the enterprises’ profits and social welfare. Therefore, the government can promote the development of the pharmaceutical industry through this strategy.

#### 4.3.2 Patient medical insurance subsidy strategy. (*CR*)

Under the patient medical insurance subsidy strategy, the (1 − *η*) is used to represent the medical insurance reimbursement ratio. The smaller *η* is, the larger the medical insurance reimbursement ratio is, where *η* ∈ [0,1]; and *pη* represents the actual price of the new drug purchased by the patients, the patient’s price sensitivity coefficient becomes *bη*, so the patient’s price demand function is $p=\frac{a}{\eta }-bQ$, using $\pi_{si}^{CR}$ and $\pi_{di}^{CR}$ to represent the profits of upstream and downstream pharmaceutical enterprises, which are respectively as follows:

$$\pi_{si}^{CR}=\left(w-c_{si}\right)q_{si}-\frac{1}{2}{rx}_{si}^{2}=\left[w-\left(c_{1}-x_{si}-v_{1}x_{sj}-\beta_{2}\left(x_{d1}+x_{d2}\right)\right)\right]q_{si}-\frac{1}{2}{rx}_{si}^{2},\;\text{where}\;i=1,2;j=3-i$$ (56)

$$\pi_{di}^{CR}=\left(p-c_{di}\right)q_{di}-\frac{1}{2}{rx}_{di}^{2}=\left[p-\left(w+c_{2}-x_{di}-v_{2}x_{dj}-\beta_{1}\left(x_{s1}+x_{s2}\right)\right)\right]q_{di}-\frac{1}{2}{rx}_{di}^{2},\;\text{where}\;i=1,2;j=3-i$$ (57)

Under the patient medical insurance subsidy strategy, social welfare is expressed as the profits of all enterprises plus consumer surplus, and then subtract the government subsidies, which is as follows:

$${SW}^{CR}=\frac{1}{2}b\eta Q^{2}+\sum_{i=1}^{2}\pi_{si}+\sum_{i=1}^{2}\pi_{di}-\left(1-\eta \right)pQ,\;\text{where}\;i=1,2$$ (58)

In the above models, the government is the leader who determines the proportion of medical insurance reimbursement for new drug; the upstream and downstream pharmaceutical enterprises are followers, who determine the level of innovation input and product pricing. Lemma 5 can be obtained by backward induction.

**Lemma 5** Under the patient medical insurance subsidy strategy, the optimal decision-making, enterprises’ profits, and social welfare of the pharmaceutical production supply chain are respectively as follows:

$$x_{si}^{CR}=\frac{12\left(\frac{a}{\eta }-c_{1}-c_{2}\right)\left(\beta_{1}-\nu_{2}+2\right)}{F},\;\text{where}\;i=1,2;j=3-i$$ (59)

$$x_{di}^{CR}=\frac{2\left(\frac{a}{\eta }-c_{1}-c_{2}\right)\left(4\beta_{2}-7\nu_{1}+11\right)}{F},\;\text{where}\;i=1,2;j=3-i$$ (60)

$$q^{CR}=\frac{\left(\frac{a}{\eta }-c_{1}-c_{2}\right)\left[2F+4\left(\nu_{2}+1+2\beta_{2}\right)\left(4\beta_{2}-7\nu_{1}+11\right)+24\left(2\beta_{1}+\nu_{1}+1\right)\left(\beta_{1}-\nu_{2}+2\right)\right]}{9bF}$$ (61)

$$w^{CR}=\frac{\left(2\frac{a}{\eta }-2c_{2}+4c_{1}\right)F+\left(\frac{a}{\eta }-c_{1}-c_{2}\right)\left[4\left(\nu_{2}+1-4\beta_{2}\right)\left(4\beta_{2}-7\nu_{1}+11\right)+24\left(2\beta_{1}-2\nu_{1}-2\right)\left(\beta_{1}-\nu_{2}+2\right)\right]}{6F}$$ (62)

$$P^{CR}=\frac{\left(5\frac{a}{\eta }+4c_{2}+4c_{1}\right)F-2\left(\frac{a}{\eta }-c_{1}-c_{2}\right)\left[4\left(2\beta_{2}+\nu_{2}+1\right)\left(4\beta_{2}-7\nu_{1}+11\right)+24\left(2\beta_{1}+\nu_{1}+1\right)\left(\beta_{1}-\nu_{2}+2\right)\right]}{9F}$$ (63)

$$\pi_{si}^{CR}=\frac{{\left[\left(\frac{a}{\eta }-c_{1}-c_{2}\right)\left[2F+4\left(\nu_{2}+2\beta_{2}+1\right)\left(4\beta_{2}-7\nu_{1}+11\right)+24\left(2\beta_{1}+\nu_{1}+1\right)\left(\beta_{1}-\nu_{2}+2\right)\right]\right]}^{2}}{54bF^{2}}-\frac{1}{2}r{\left[\frac{12\left(\frac{a}{\eta }-c_{1}-c_{2}\right)\left(\beta_{1}-\nu_{2}+2\right)}{F}\right]}^{2}$$ (64)

$$\pi_{di}^{CR}=\frac{{\left[\left(\frac{a}{\eta }-c_{1}-c_{2}\right)\left[2F+4\left(\nu_{2}+2\beta_{2}+1\right)\left(4\beta_{2}-7\nu_{1}+11\right)+24\left(2\beta_{1}+\nu_{1}+1\right)\left(\beta_{1}-\nu_{2}+2\right)\right]\right]}^{2}}{81bF^{2}}-\frac{1}{2}r{\left[\frac{2\left(\frac{a}{\eta }-c_{1}-c_{2}\right)\left(4\beta_{2}-7\nu_{1}+11\right)}{F}\right]}^{2}$$ (65)

$$\begin{array}{l} {SW}^{CR}=\frac{14{\left[\left(\frac{a}{\eta }-c_{1}-c_{2}\right)\left[2F+4\left(\nu_{2}+2\beta_{2}+1\right)\left(4\beta_{2}-7\nu_{1}+11\right)+24\left(2\beta_{1}+\nu_{1}+1\right)\left(\beta_{1}-\nu_{2}+2\right)\right]\right]}^{2}}{162bF^{2}}\\ -\frac{162br{\left(\frac{a}{\eta }-c_{1}-c_{2}\right)}^{2}\left[144{\left(\beta_{1}-\nu_{2}+2\right)}^{2}+4{\left(4\beta_{2}-7\nu_{1}+11\right)}^{2}\right]}{162bF^{2}}-\left(1-\eta \right)•\frac{\left(5\frac{a}{\eta }+4c_{2}+4c_{1}\right)F-2\left(\frac{a}{\eta }-c_{1}-c_{2}\right)G}{9F}•\frac{2\left(\frac{a}{\eta }-c_{1}-c_{2}\right)\left[2F+G\right]}{9bF} \end{array}$$ (66)

The proof process is similar to Lemma 1, it will not be repeated here.

**Corollary 8** Under the patient medical insurance subsidy strategy, the impact of subsidies on pharmaceutical enterprises’ decision-making and social welfare are respectively as follows:

(i)
$\frac{\partial x_{si}^{CR}}{\partial \left(1-\eta \right)}>0,\frac{\partial x_{di}^{CR}}{\partial \left(1-\eta \right)}>0,\;\text{where}\;i=1,2$

(ii)
$\frac{\partial q^{CR}}{\partial \left(1-\eta \right)}>0$

(iii)
$\frac{\partial w^{CR}}{\partial \left(1-\eta \right)}>0,\frac{\partial p^{CR}}{\partial \left(1-\eta \right)}>0$

(iv)
$\frac{\partial \pi_{si}^{CR}}{\partial \left(1-\eta \right)}>0,\frac{\partial \pi_{si}^{CR}}{\partial \left(1-\eta \right)}>0$

(v)
$\frac{\partial {SW}^{ME}}{\partial \left(1-\eta \right)}<0$

Corollary 8 (i) has shown that as the government reimburses new drug with a greater proportion of medical insurance, all enterprises will increase their investment in new drug R&D. From (ii) and (iii), it can be seen that as the proportion of medical insurance reimbursements for new drug increases, the number of patients choosing new drugs will increase. Meanwhile, under the influence of medical insurance, as demand increases, these enterprises will increase the prices of intermediate product and new drug. From (iv) and (v), it can be seen that under the influence of both price and demand, the profits of enterprises will increase. The government pays for the new drug for patients, and the medical insurance expenditure is greater than consumer surplus and the enterprises’ profit growth. Therefore, social welfare will decrease as the proportion of medical insurance reimbursement increases. In summary, under the patient medical insurance subsidy strategy, although the social welfare will be reduced, the accessibility of patients was improved, the profits of enterprises could also be improved, and the new drug R&D of pharmaceutical enterprises was promoted.

**Corollary 9** According to the maximization of social welfare, taking the derivative of *SW*^*CR*^ with respect to *η*, and set $\frac{\partial {SW}^{CR}}{\partial \eta }=0$, then it is obtained as follows:

$$\eta =\sqrt[{3}]{\frac{9\left[\left(8F+22G\right)\left(2F+G\right)+162br\left[144{\left(\beta_{1}-\nu_{2}+2\right)}^{2}+4{\left(4\beta_{2}-7\nu_{1}+11\right)}^{2}\right]\right]}{2a\left(c_{1}+c_{2}\right)\left[\left(26F+22G\right)\left(2F+G\right)-162br\left[144{\left(\beta_{1}-\nu_{2}+2\right)}^{2}+4{\left(4\beta_{2}-7\nu_{1}+11\right)}^{2}\right]\right]}}$$ (67)

Then analyzing the impact of the horizontal spillover effects and the vertical spillover effects on the new drug medical insurance reimbursement ratio (1 − *η*), which is as follows:

$$\frac{\partial \left(1-\eta \right)}{\partial \nu_{1}}>0,\frac{\partial \left(1-\eta \right)}{\partial v_{2}}>0;\frac{\partial \left(1-\eta \right)}{\partial \beta_{1}}<0,\frac{\partial \left(1-\eta \right)}{\partial \beta_{2}}<0$$

It can be seen from Corollary 9 that under the patient medical insurance subsidy strategy, the new drug medical insurance reimbursement ratio (1 − *η*) increases as the horizontal spillover rate *ν* increases, and decreases as the vertical spillover rate *β* increases. It shows that spillover effects can affect the reimbursement ratio of new drug insurance, so it is necessary for the government to consider the spillover effects of enterprise new drug R&D when formulating new drug medical insurance strategy.

**Proposition 5** Comparing the non-government subsidy strategy and patient medical insurance subsidy strategy, which can be obtained as follows:

$$x_{si}^{CR}>x_{si}^{B},x_{di}^{CR}>x_{di}^{B},\pi_{si}^{CR}>\pi_{si}^{B},\pi_{di}^{CR}>\pi_{di}^{B},{SW}_{di}^{CR}>{SW}_{di}^{B}.$$

It can be seen from Proposition 5 that compared with the non-government subsidy strategy, the patient medical insurance subsidy strategy has a positive incentive effect on new drug R&D of pharmaceutical enterprises, which helps to increase the enterprises’ profits and social welfare. Therefore, the government can promote the development of the pharmaceutical industry through patient medical insurance subsidies.

## 5 Comparison of equilibrium solutions of five models

Market economy has led to imperfect innovation results, and government subsidy intervention can improve this market failure to a certain extent. But enterprises’ R&D spillover effects have a greater impact on government subsidies. In order to better analyze the impact of double spillover effects on government subsidies, and the impact of subsidies on the decision-making and social welfare of the pharmaceutical production supply chain. It is assumed that the horizontal spillovers within the upstream and downstream pharmaceutical enterprises are equal, that is *ν*_1_ = *ν*_2_ = *ν*, and the vertical spillovers between these enterprises are also equal, that is *β*_1_ = *β*_2_ = *β*. In view of the fact that the enterprises pursue maximization of their own profit, while the government pursues the maximization of social welfare, next, analyzing and compares the profits and social welfare of pharmaceutical enterprises under the five subsidy strategies.

### 5.1 Profit comparison of pharmaceutical enterprises

Comparing the profits of pharmaceutical enterprises under different government subsidy strategies, which can be obtained as follows:

(i) If 0 < *ν* < *L*_3_ or 0 < *β* < *L*_4_,
  then $\pi_{si}^{CR}>\pi_{si}^{CI}=\pi_{si}^{ME}>\pi_{si}^{MS}>\pi_{si}^{B},\pi_{di}^{CR}>\pi_{di}^{CI}=\pi_{di}^{ME}>\pi_{di}^{MS}>\pi_{di}^{B};\;\text{where}\;i\;=1,2$;
  If *ν* < *L*_3_ or *β* < *L*_4_,
  then $\pi_{si}^{CR}>\pi_{si}^{CI}=\pi_{si}^{ME}>\pi_{si}^{B}{>\pi }_{si}^{MS},\pi_{di}^{CR}>\pi_{di}^{CI}=\pi_{di}^{ME}>\pi_{di}^{B}>\pi_{di}^{MS};\;\text{where}\;\;i=1,2$;
(ii)
$\begin{array}{l} \frac{\partial \pi_{si}^{CR}}{\partial \nu }>\frac{\partial \pi_{si}^{ME}}{\partial \nu }=\frac{\partial \pi_{si}^{CI}}{\partial \nu }>\frac{\partial \pi_{si}^{B}}{\partial \nu }>0;\frac{\partial \pi_{di}^{CR}}{\partial \nu }>\frac{\partial \pi_{di}^{ME}}{\partial \nu }=\frac{\partial \pi_{di}^{CI}}{\partial \nu }>\frac{\partial \pi_{di}^{B}}{\partial \nu }>0,\;\text{where}\;i=1,2\\ \frac{\partial \pi_{si}^{ME}}{\partial \beta }=\frac{\partial \pi_{si}^{CI}}{\partial \beta }>\frac{\partial \pi_{si}^{B}}{\partial \beta }>\frac{\partial \pi_{si}^{CR}}{\partial \beta }>0;\frac{\partial \pi_{di}^{ME}}{\partial \beta }=\frac{\partial \pi_{di}^{CI}}{\partial \beta }>\frac{\partial \pi_{di}^{B}}{\partial \beta }>\frac{\partial \pi_{di}^{CR}}{\partial \beta }>0,\;\text{where}\;i=1,2 \end{array}$

(iii) If 0 < *ν* < *L*_1_ or 0 < *β* < *L*_2_, then $\frac{\partial \pi_{si}^{ME}}{\partial \nu }>0,\frac{\partial \pi_{di}^{ME}}{\partial \nu }>0;\frac{\partial \pi_{si}^{ME}}{\partial \beta }>0,\frac{\partial \pi_{di}^{ME}}{\partial \beta }>0$;
  If *ν* > *L*_1_ or *β* > *L*_2_,then $\frac{\partial \pi_{si}^{ME}}{\partial \nu }<0,\frac{\partial \pi_{di}^{ME}}{\partial \nu }<0;\frac{\partial \pi_{si}^{ME}}{\partial \beta }<0,\frac{\partial \pi_{di}^{ME}}{\partial \beta }<0$.

By comparison, (i) shows that when the spillover effects are small, the government subsidy strategies can increase the enterprises’ profits. Among them, the upstream and downstream pharmaceutical enterprises under the patient medical insurance subsidy strategy have the highest profits, followed by the patient price subsidy strategy and the pharmaceutical enterprise innovative product subsidy strategy, then followed by the pharmaceutical enterprises’ innovation input subsidy strategy. When the spillover effects are large, the profit of pharmaceutical enterprises under the subsidy of innovation input will change, which will be lower than the profit under the strategy of non-government subsidy. The main reason for the highest enterprise profit under the patient medical insurance subsidy strategy is that the target of the subsidy is the patient, and it belongs to the ex-post subsidy mode of payment before reimbursement, so this subsidy is more intuitive; besides this subsidy strategy can reduce the patient’s price sensitivity coefficient, so it has the highest demand; meanwhile, under the patient medical insurance subsidy strategy, the product pricing of pharmaceutical enterprises will be higher than the product pricing under the enterprises subsidy strategy. Under the influence of multiple factors, enterprises have the highest profits under this type of subsidy strategy. The main reason why the profit of pharmaceutical enterprises under the innovation input subsidy strategy has increased firstly and then decreased as the spillover effect becomes larger is that when the spillover effect is large, enterprises rely too much on technology spillovers from their peers and do not pay attention to their own R&D investment. This kind of "free rider" mentality is not conducive to the development of enterprises.

(ii) and (iii) show that when the spillover effects are small, with the increase of the horizontal spillover effects and the vertical spillover effects, the profits of the pharmaceutical enterprises can be improved. when the spillover effects are large, the profits of pharmaceutical enterprises under the innovation input subsidy strategy decreases with the increase of spillover effects, while the enterprises’ profits under the other subsidy strategies increases with the increase of spillover effects.

### 5.2 Comparison of social welfare

Comparing social welfare under different government subsidy strategies, which are respectively as follows:

(i)
${SW}^{CR}>{SW}^{CI}={SW}^{ME}>{SW}^{MS}>{SW}^{B}$

(ii)
$\frac{\partial {SW}^{CR}}{\partial \nu }>\frac{\partial {SW}^{ME}}{\partial \nu }=\frac{\partial {SW}^{CI}}{\partial \nu }>\frac{\partial {SW}^{MS}}{\partial \nu }>\frac{\partial {SW}^{B}}{\partial \nu }>0$

(iii)
$\frac{\partial {SW}^{ME}}{\partial \beta }=\frac{\partial {SW}^{CI}}{\partial \beta }>\frac{\partial {SW}^{CR}}{\partial \beta }>\frac{\partial {SW}^{MS}}{\partial \beta }>\frac{\partial {SW}^{B}}{\partial \beta }>0$

By comparison, (i) shows that the government can improve social welfare through subsidies, and under the same spillover effects, the patient medical insurance subsidy strategy can significantly improve social welfare. This is mainly because this subsidy strategy can better consider the intuitive feelings of patients, and guides patients to purchase new drug through medical insurance reimbursement, which not only increases consumer surplus, but also promotes the increase in the production capacity of pharmaceutical enterprises and increases their profits.

(ii) shows that the horizontal spillover effects have positive incentive effect on social welfare under different subsidy strategies, and the impact on social welfare under the patient medical insurance subsidy strategy is the most significant. This is mainly because as the increases of horizontal spillover effects, the production cost of pharmaceutical companies is relatively reduced, and product pricing is also reduced, and patient demand increases; in addition, the reduction in product pricing will reduce government medical insurance subsidies. Under multiple influences, the social welfare under the patient medical insurance subsidy strategy will be increased significantly.

(iii) shows that the vertical spillover effects have positive incentive effect on social welfare under different subsidy strategies, and the impact on social welfare under the pharmaceutical enterprise innovative product subsidy strategy is the most significant. This is mainly because under the influence of vertical spillover effects, all enterprises have significantly reduced product pricing, increasing consumer demand and enterprises’ profits and social welfare.

In summary, through the above analysis of the decision-making behavior of pharmaceutical enterprises and the government, it is obvious that whether it is from the perspective of pharmaceutical enterprises profits or from the perspective of social welfare, the patient subsidy strategies are better than the pharmaceutical enterprises subsidy strategies, Among them, the medical insurance subsidy strategy is the most significant. This just overturns a view of traditional incentive theory: traditional incentive theory believes that ex-ante subsidy strategies are better than ex-post subsidy strategies. As for subsidy objects, the pharmaceutical enterprises subsidy strategies are ex-ante subsidy strategies, and the patient subsidy strategies are ex-post subsidy strategies. As for subsidy methods, the pharmaceutical enterprise innovation input subsidy strategy is ex-ante subsidy, and the pharmaceutical enterprise innovative product subsidy strategy is ex-post subsidy. In today’s society where innovation and technology are becoming increasingly complex, the R&D investment is increasing, the risk of R&D and the failure rate are higher. The government’s ex-ante subsidy strategy cannot effectively stimulate enterprises to increase R&D investment for higher returns. However, under the ex-post subsidy strategy, enterprises can obtain corresponding subsidies and benefits after successful R&D, which can further stimulate their R&D enthusiasm, forming a virtuous circle. The government’s ex-post incentive strategy leads to higher enterprise output, promotes more effective technological innovation, and enables enterprises to produce greater innovation results with less innovation input, which accordingly leads to a better level of social welfare.

## 6 Numerical analysis

In order to illustrate the impact of the double spillover effects on the government subsidy strategy in the new drug R&D in pharmaceutical industry. Assuming that *a* = 4, *c*_1_ = 1, *c*_2_ = 1, *r* = 3, *b* = 1. At the same time, a single variable method is adopted and to assume that *ν* = 0.1, *β* = 0.1, after the numerical calculation, the changes of pharmaceutical enterprises and government’s decisions with spillover effects under different government subsidy strategies can be obtained.

**Fig 1:**
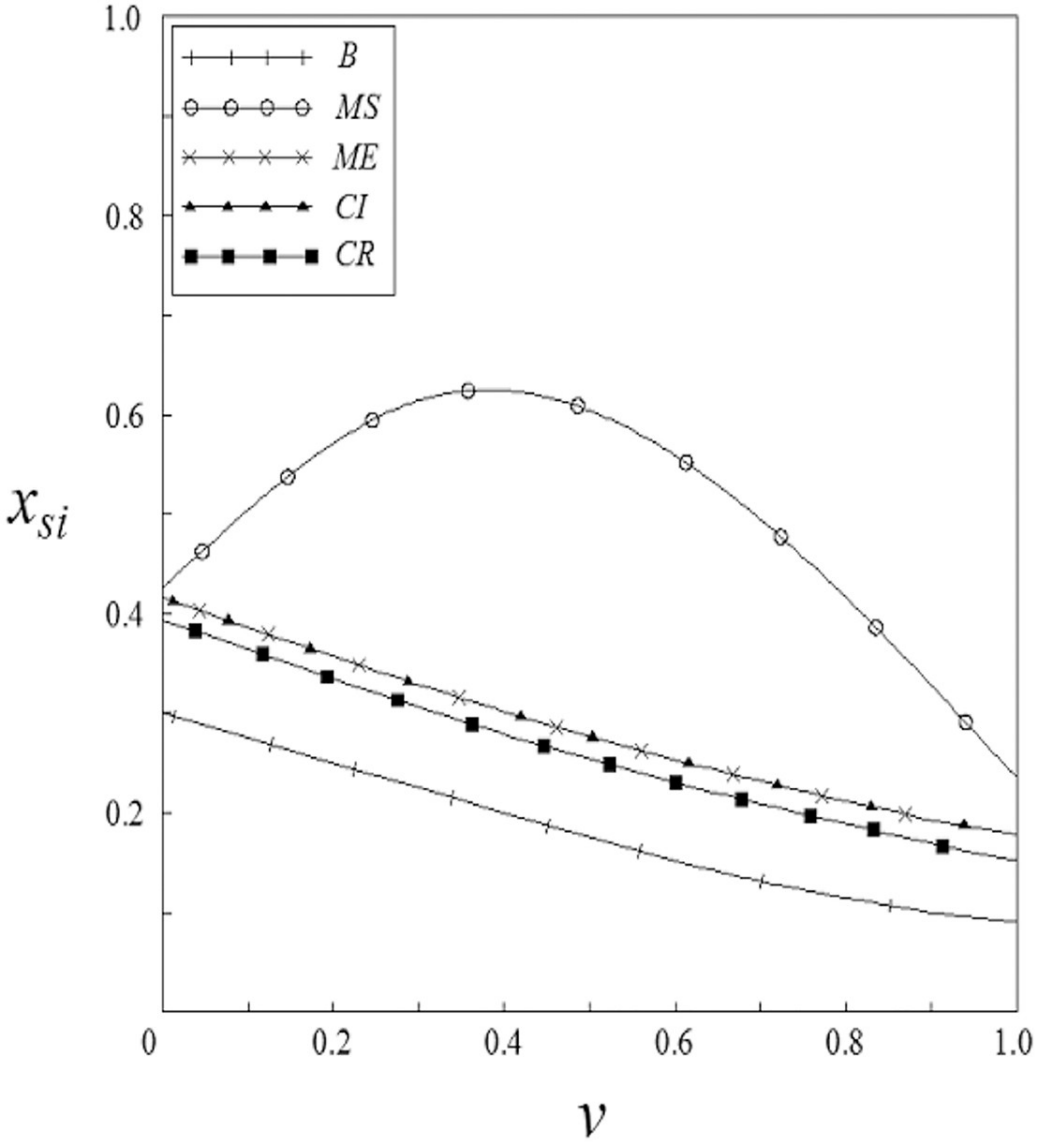
The relationship between *x*_*si*_ and *ν*.

**Fig 2:**
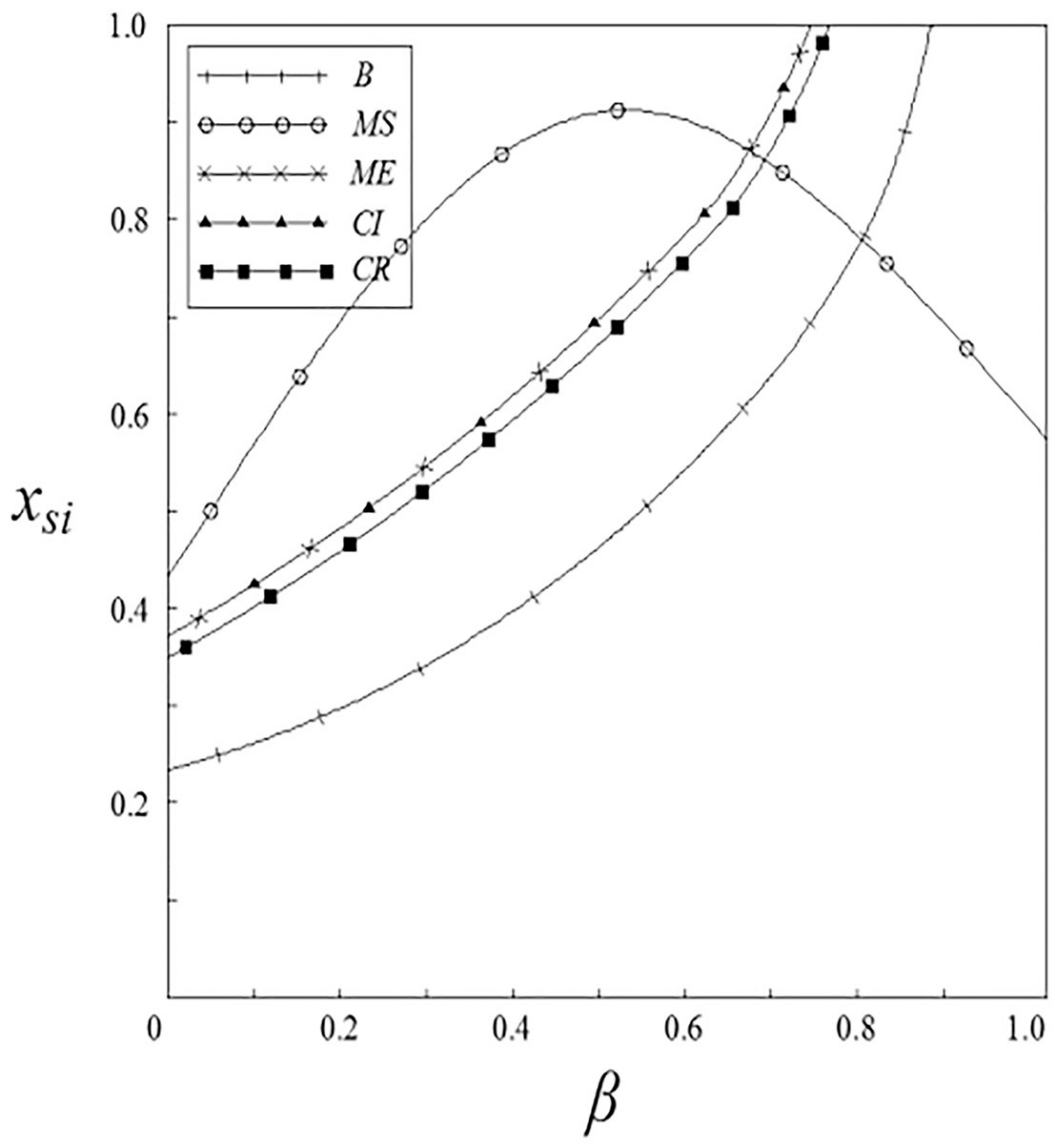
The relationship between *x*_*si*_ and *β*.

**Fig 3:**
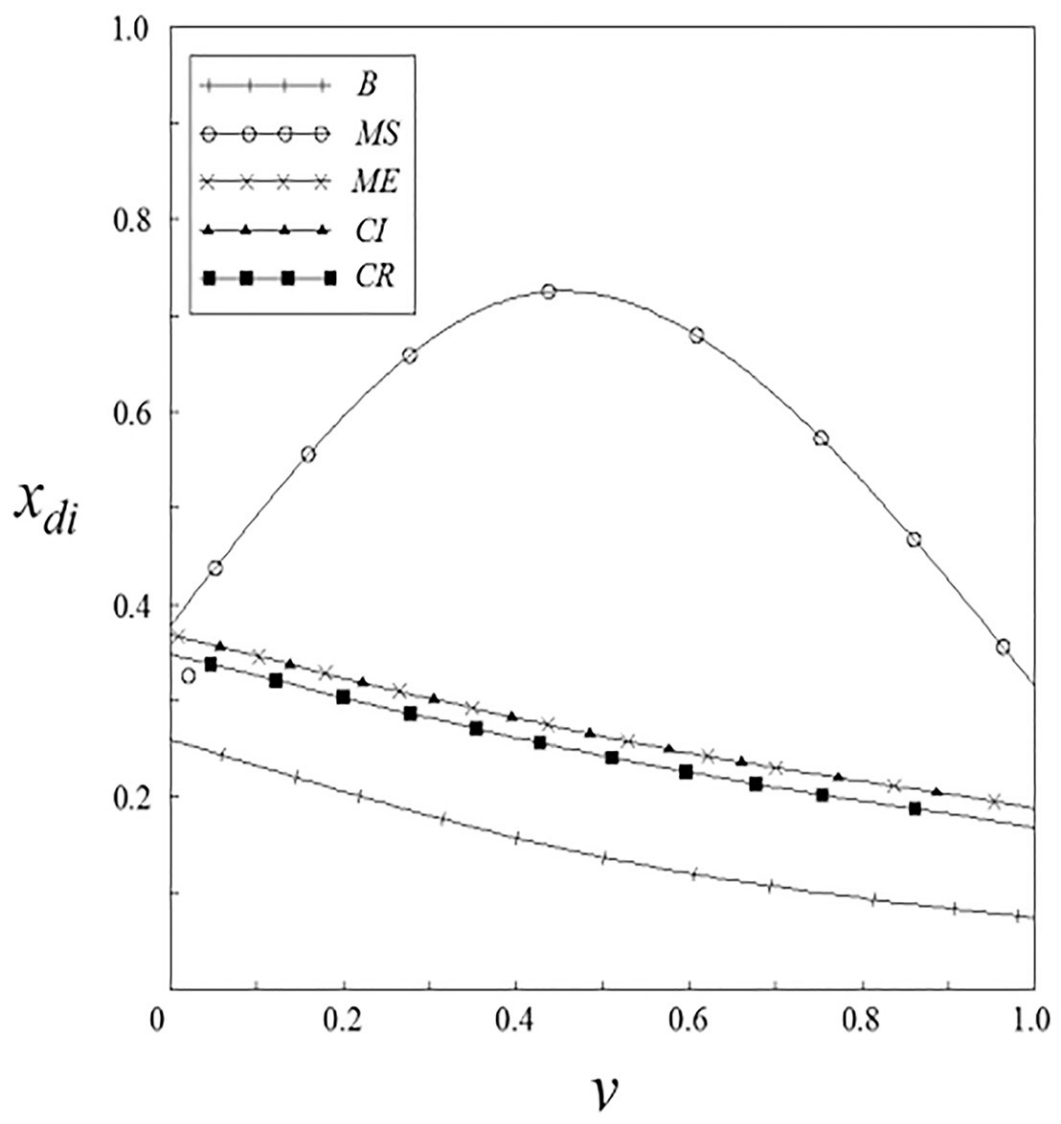
The relationship between *x*_*di*_ and *ν*.

**Fig 4:**
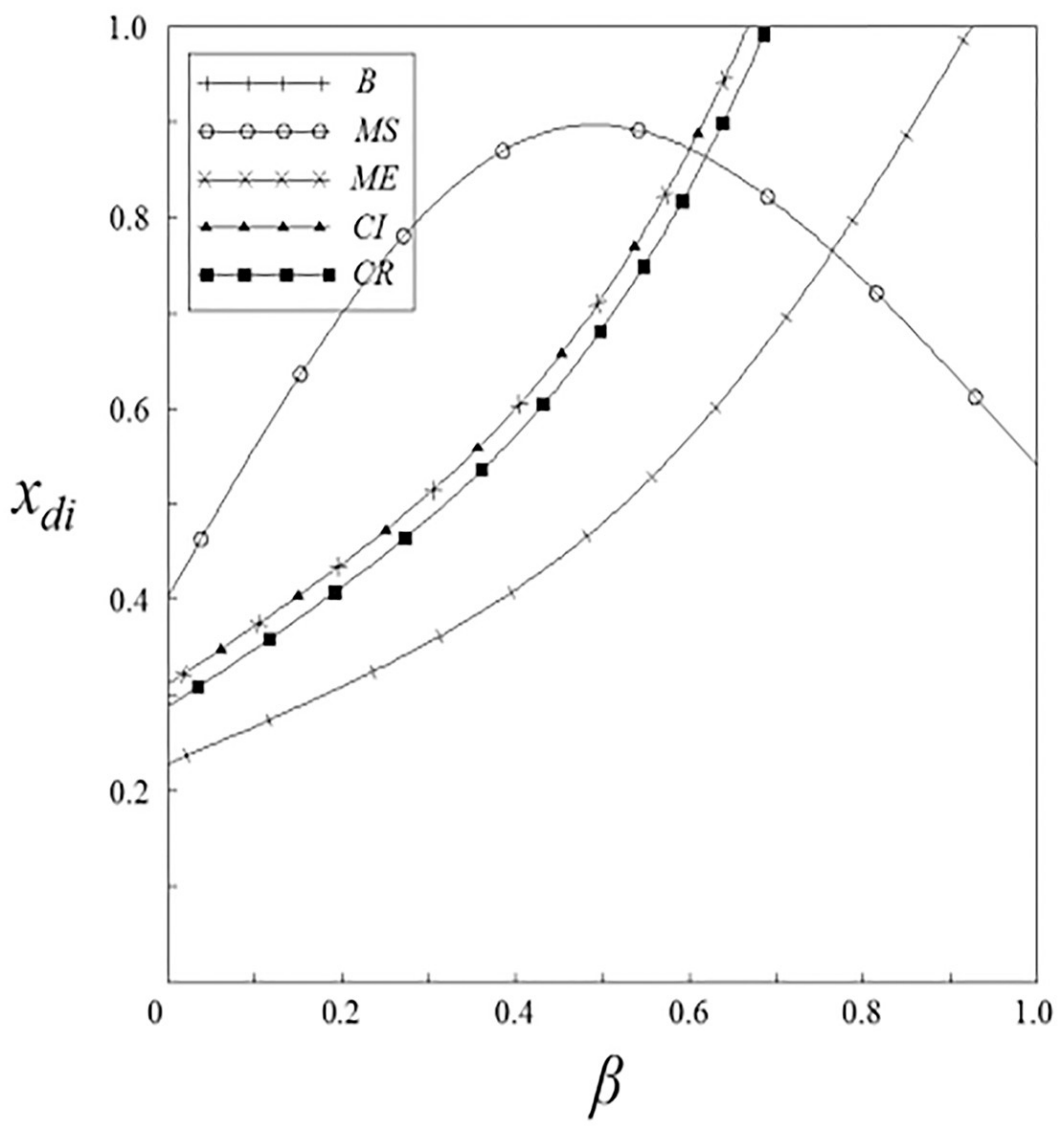
The relationship between *x*_*di*_ and *β*.

Figs 1–4 have shown that the R&D investment of upstream and downstream pharmaceutical enterprises is affected by the double spillover effects. It can be seen from these figures that under the pharmaceutical enterprises’ innovation input subsidy strategy, when the spillover effects are small, the R&D investment increases with the increase of the spillover effects; when the spillover effects are large, the R&D investment decreases with the increase of the spillover effects. The R&D investment under the remaining subsidy strategies decreases with the increase of horizontal spillover effects, and increases with the increase of vertical spillover effects.

**Fig 5:**
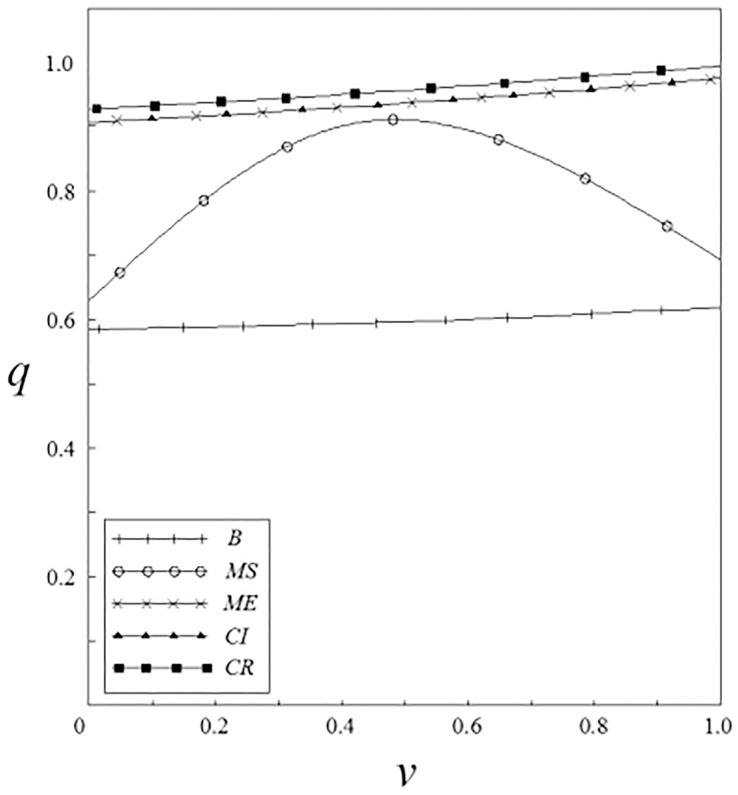
The relationship between *q* and *ν*.

**Fig 6:**
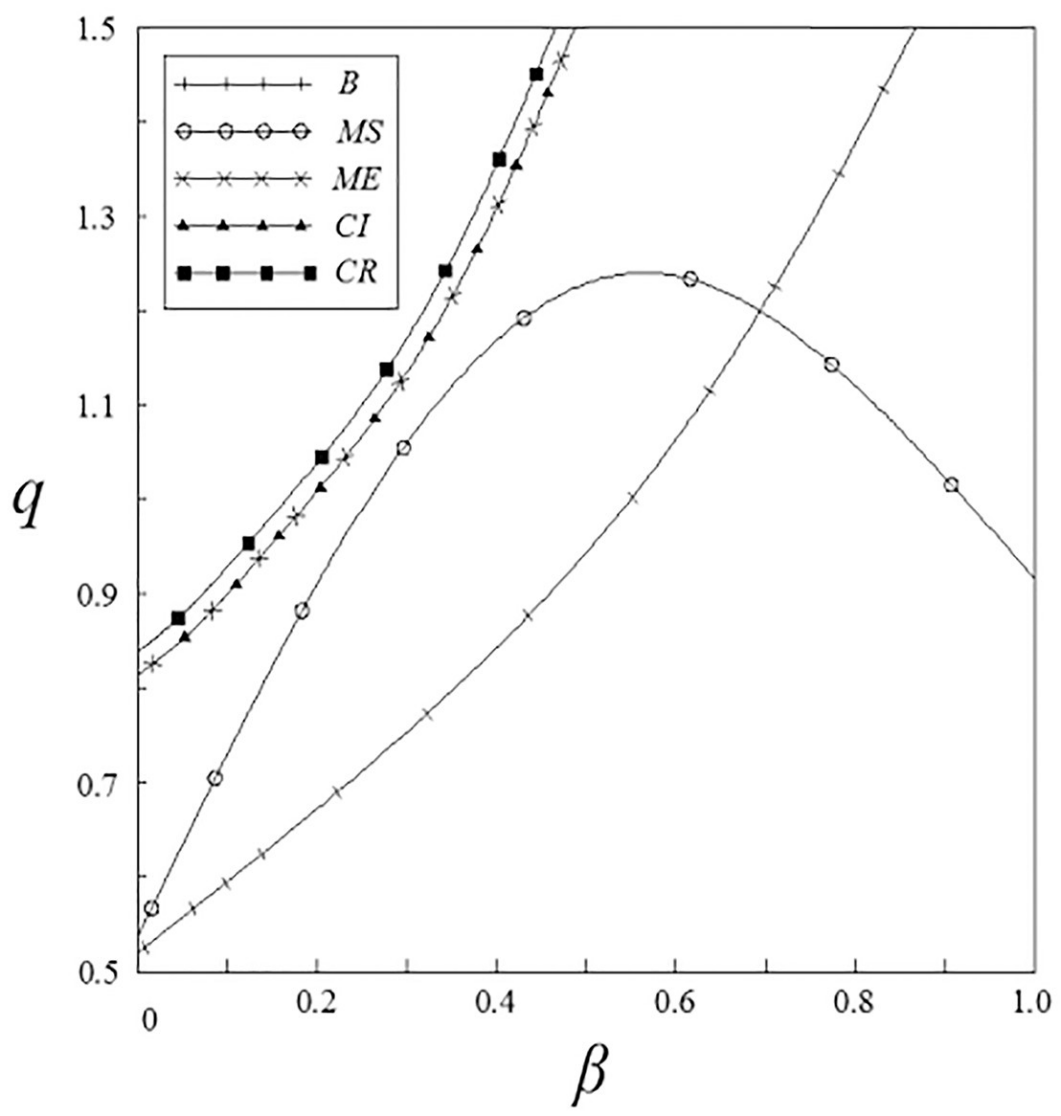
The relationship between *q* and *β*.

Figs 5 and 6 have shown that enterprises’ output is affected by spillover effects. No matter what subsidy strategy the government adopts, the output of pharmaceutical enterprises is higher than that under the non-government subsidy strategy. Among them, the output of enterprises under the pharmaceutical enterprises innovation input subsidy strategy shows a trend of firstly increase and then decrease with the increase of the spillover effects. This is related to the enterprises’ R&D investment under this subsidy strategy.

**Fig 7:**
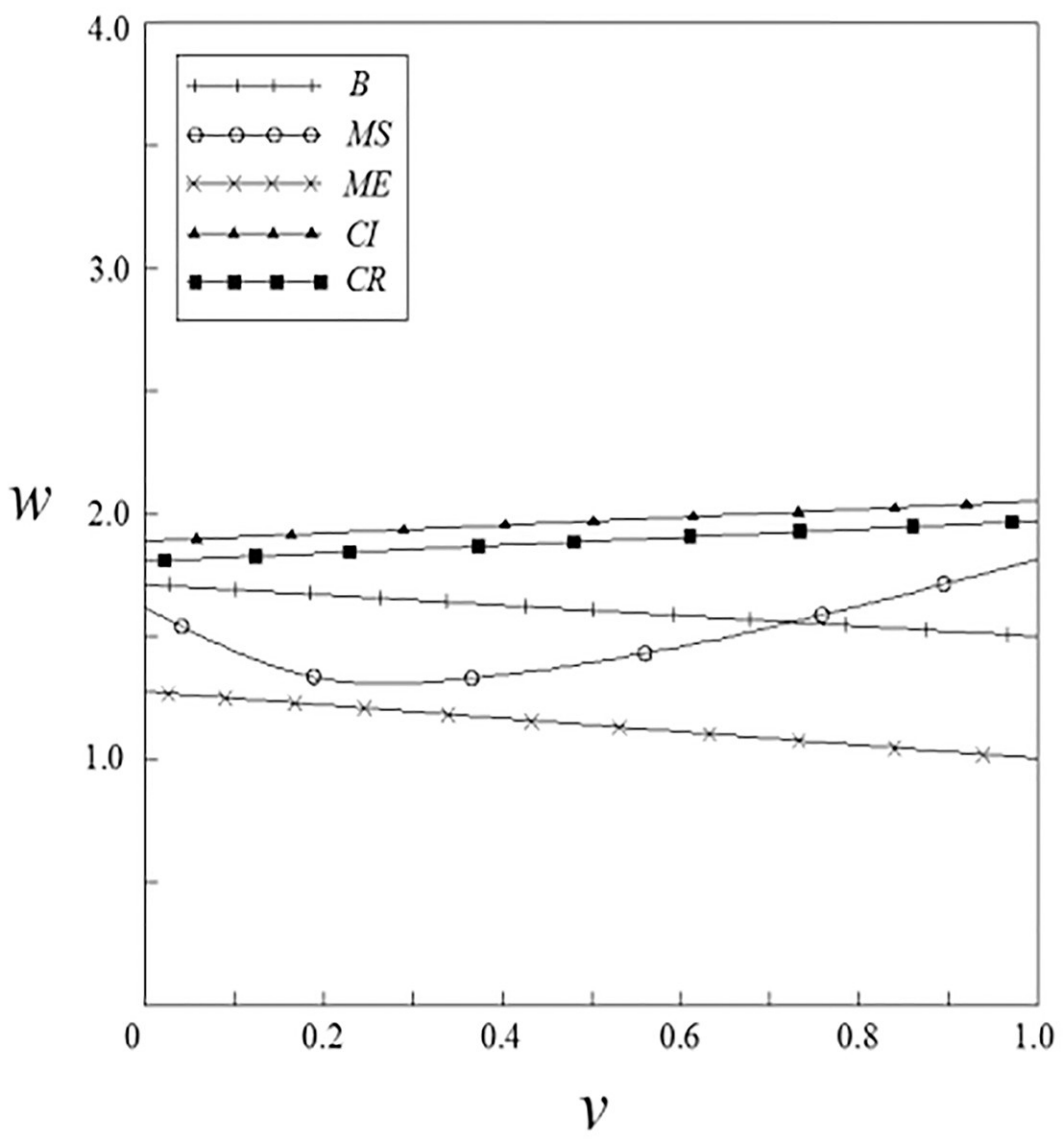
The relationship between *w* and *ν*.

**Fig 8:**
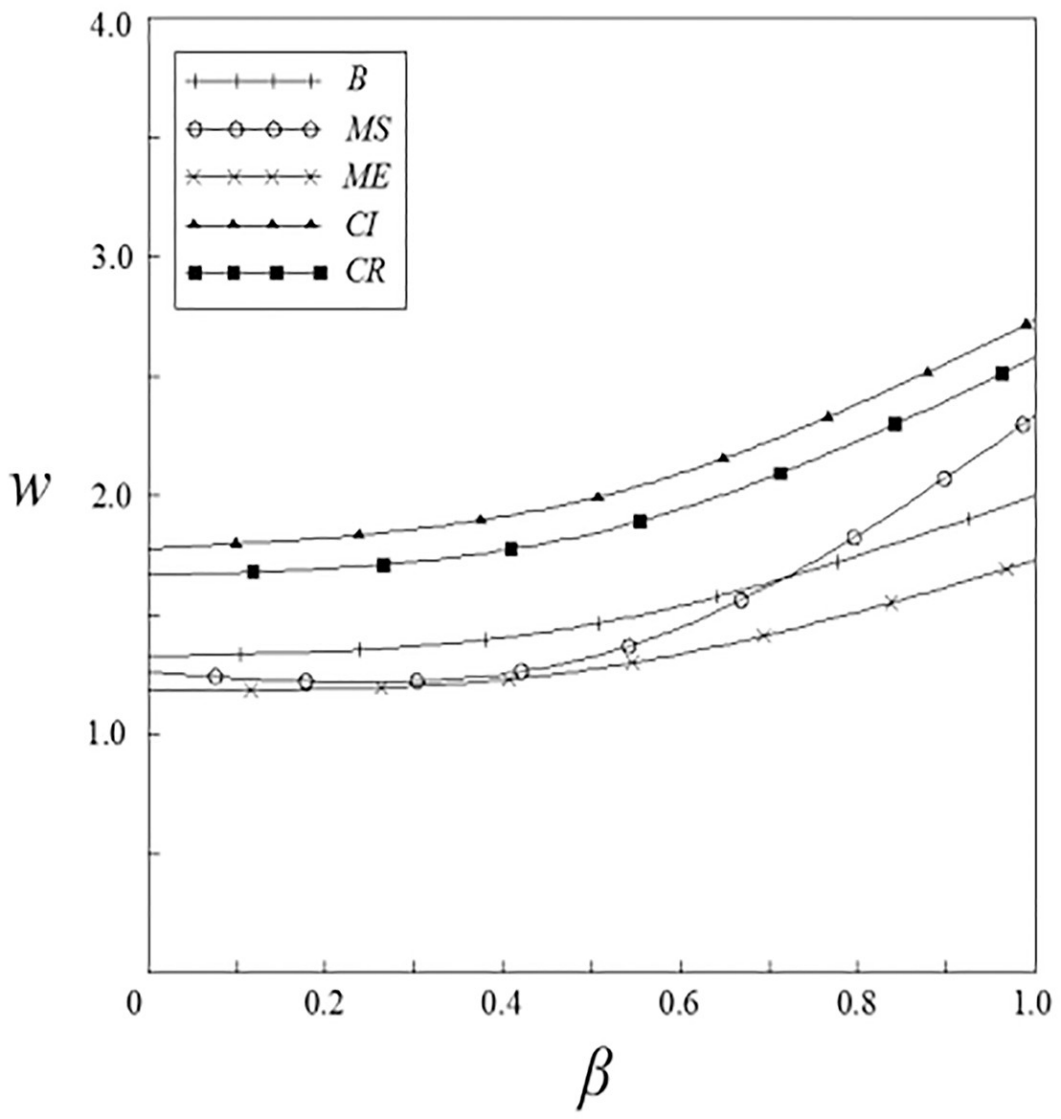
The relationship between *w* and *β*.

**Fig 9:**
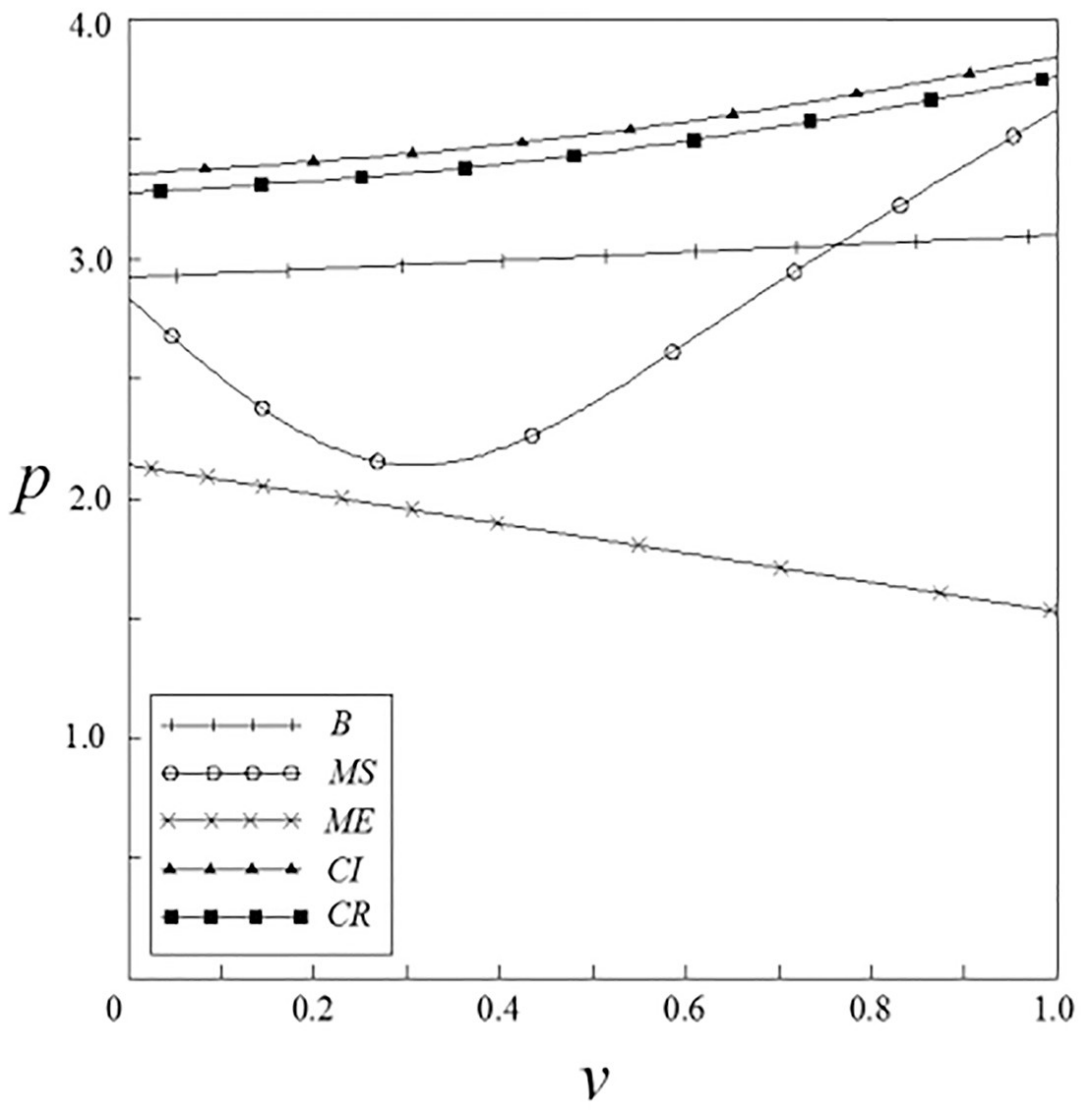
The relationship between *p* and *ν*.

**Fig 10:**
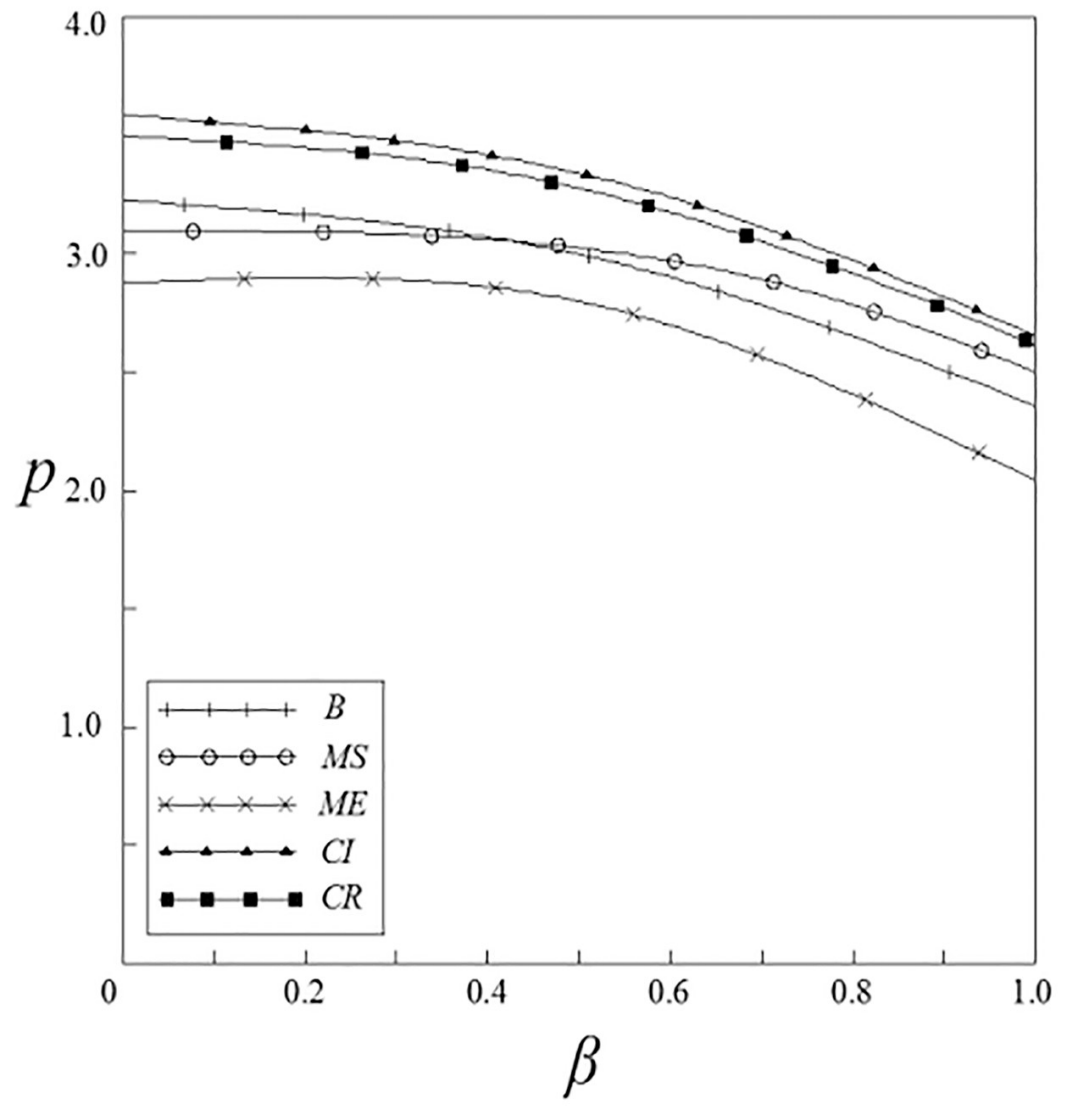
The relationship between *p* and *β*.

Figs 7–10 have shown the impact of spillover effects on enterprises pricing strategies. It can be seen from these figures that the product pricing under the patient subsidy strategy is higher than the product pricing under the non-government subsidy strategy. In the pharmaceutical enterprises subsidy strategies, the product pricing under the pharmaceutical enterprise innovative product subsidy strategy is lower than that under the non-government subsidy strategy; while the product pricing under the innovative input subsidy strategy has two situations: when the spillover effects are small, the product pricing is lower than that under the non-government subsidy strategy; When the spillover effects are large, the product pricing is higher than that under the non-government subsidy strategy, and it will increase with the increase of the spillover effects. On the whole, the pricing of products under the patient subsidy strategies are higher than that under the pharmaceutical enterprises subsidy strategies.

**Fig 11:**
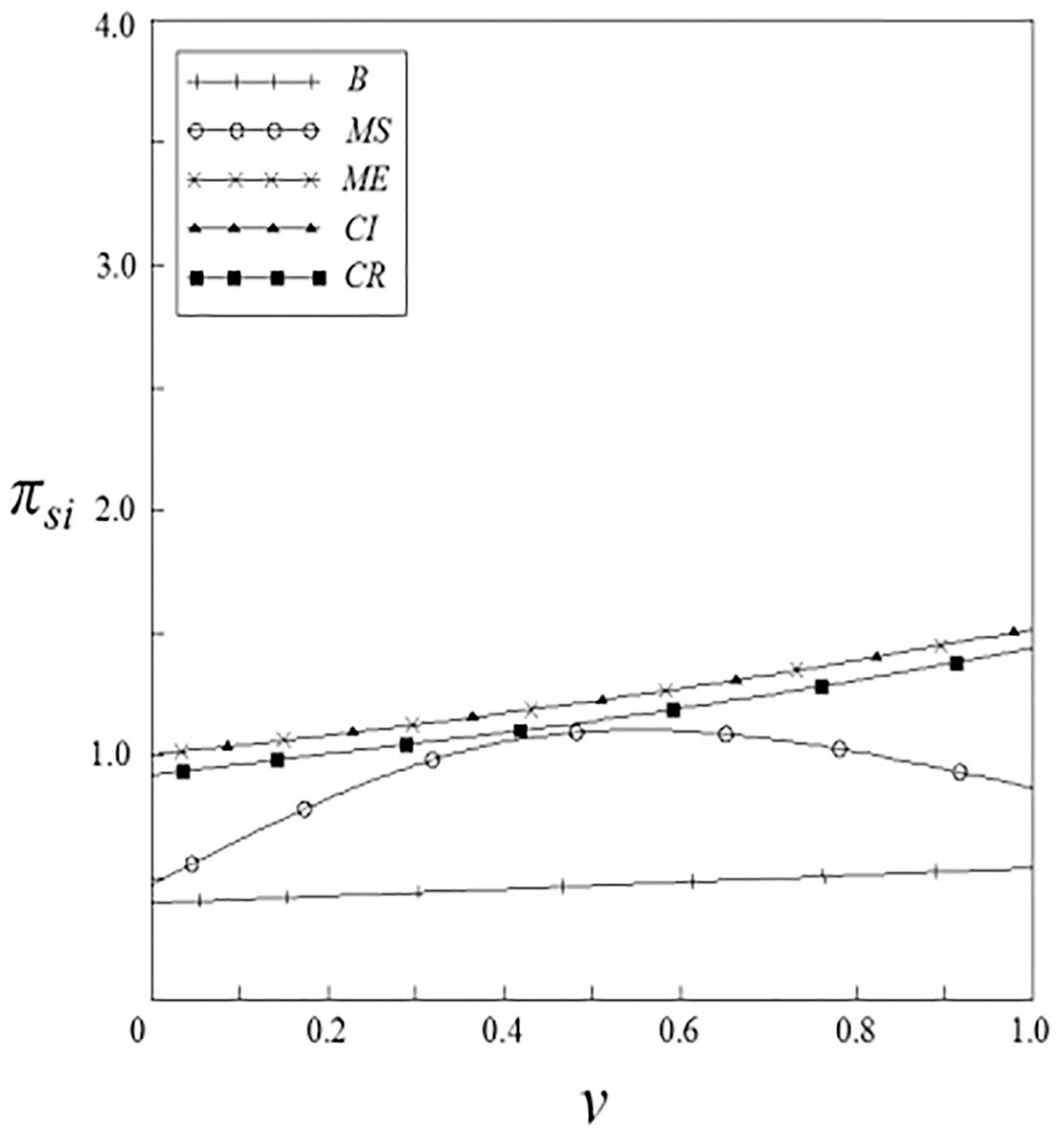
The relationship between *π*_*si*_ and *ν*.

**Fig 12:**
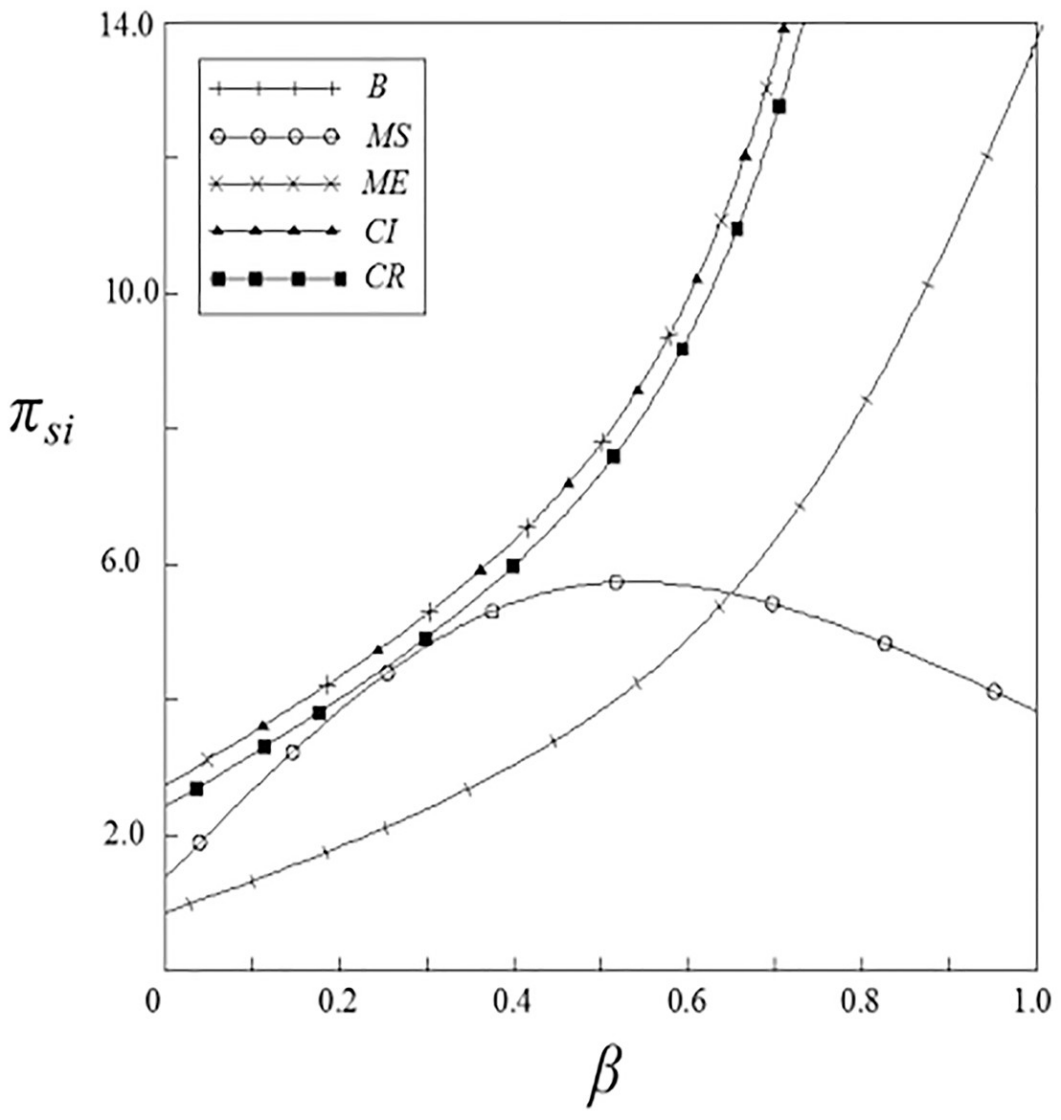
The relationship between *π*_*si*_ and *β*.

**Fig 13:**
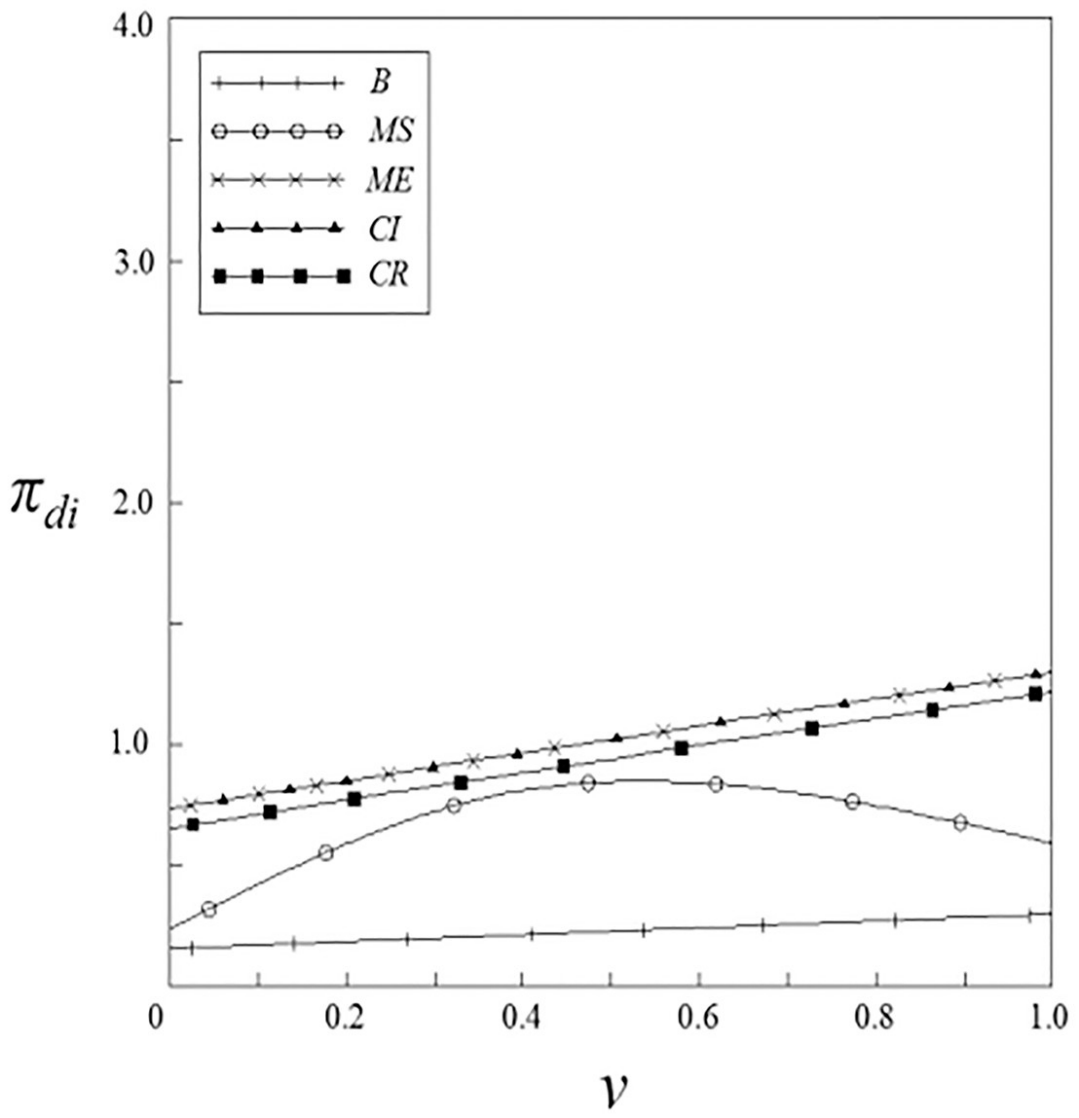
The relationship between *π*_*di*_ and *ν*.

**Fig 14:**
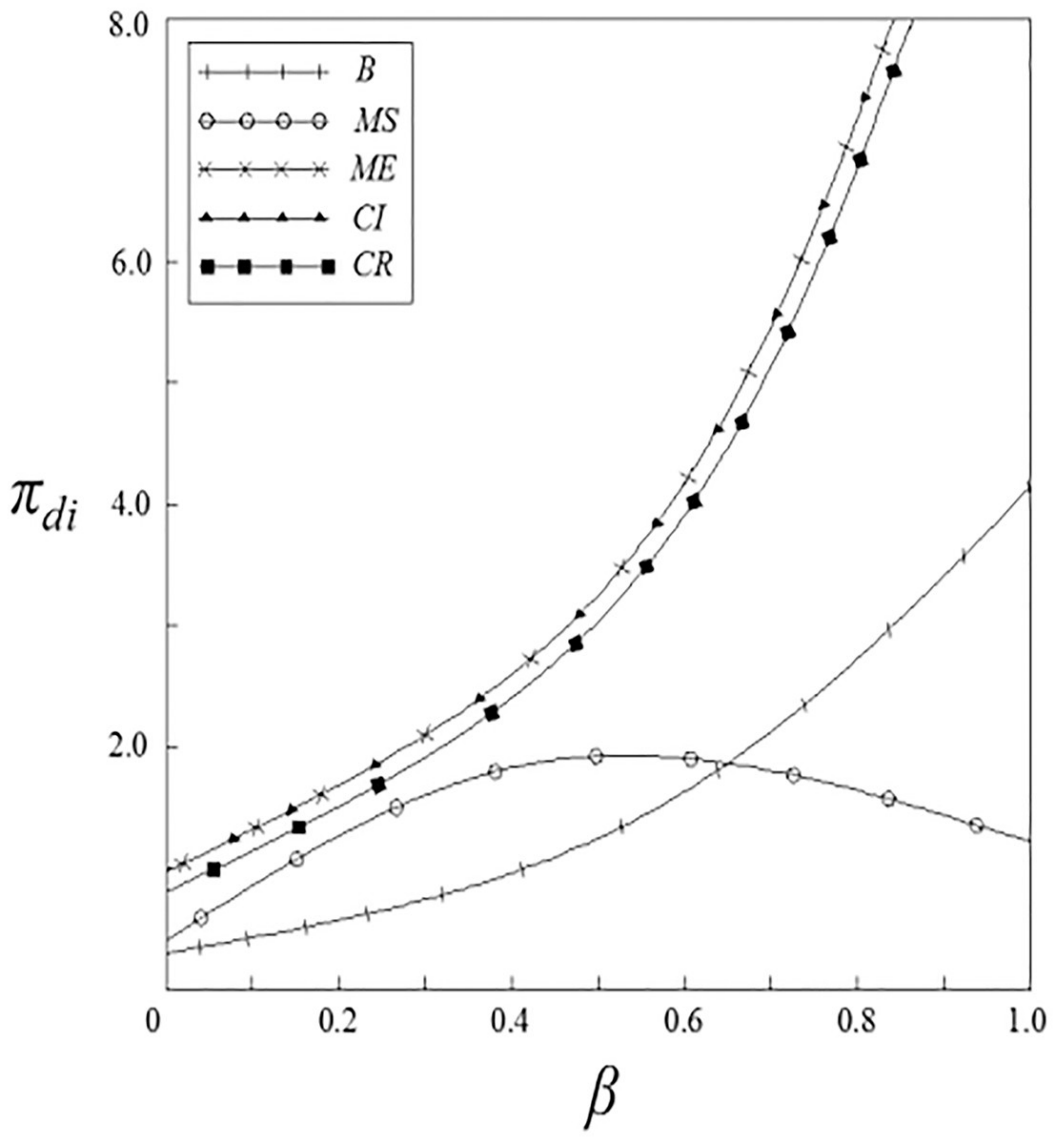
The relationship between *π*_*di*_ and *β*.

Figs 11–14 have shown the impact of spillover effects on the profits of upstream and downstream pharmaceutical enterprises. It can be seen from these figures that enterprises’ profits under the government subsidy strategies are higher than that under the non-government subsidy strategy, and the enterprise’ profits under the pharmaceutical enterprise innovative product subsidy strategy and the patient price subsidy strategy are the same. Except for the innovation input subsidy strategy, the profits under the other subsidy strategies increase with the increase of spillover effects, and the impact of vertical spillover effect on enterprises profits is more significant than horizontal spillover effect. The profit of enterprises under the innovation input subsidy strategy shows a trend of firstly increase and then decrease with the increase of the spillover effect, but the overall profit is higher than that under the non-government subsidy strategy.

**Fig 15:**
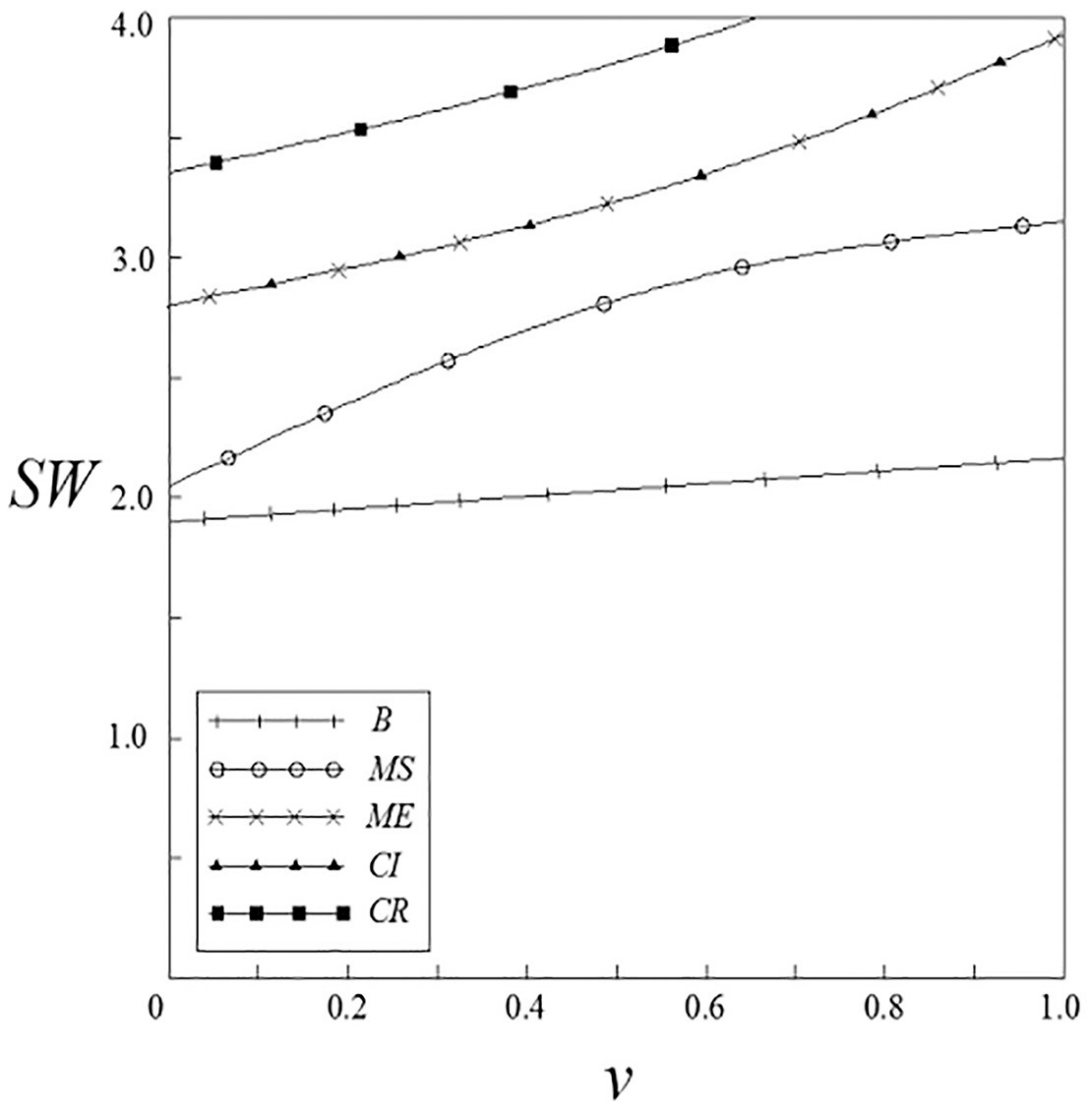
The relationship between *SW* and *ν*.

**Fig 16:**
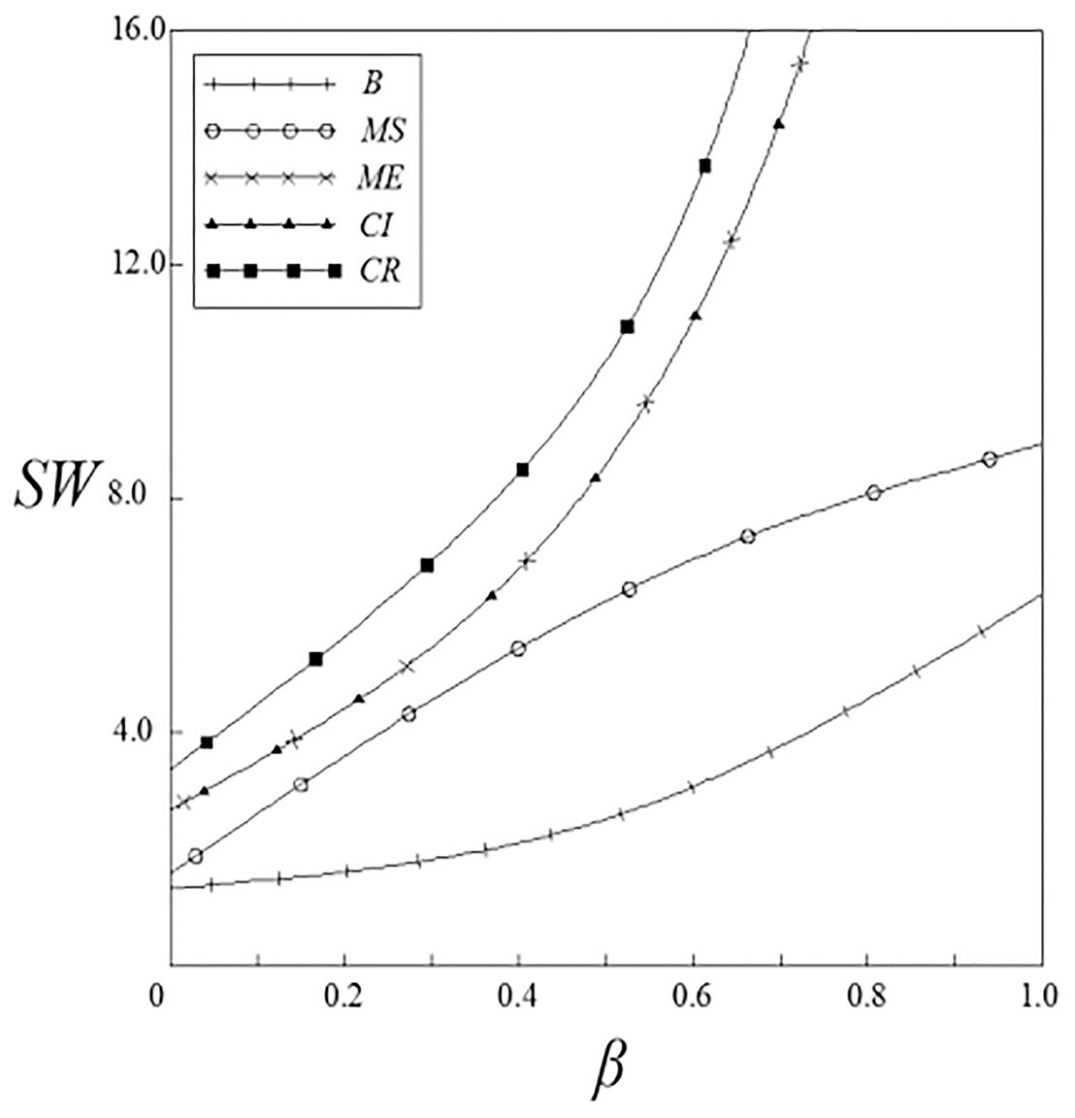
The relationship between *SW* and *β*.

Figs 15 and 16 have shown the impact of spillover effects on social welfare. It can be seen from these figures that except for the pharmaceutical enterprise innovation input subsidy strategy, the social welfare under other subsidy strategies are higher than that under the non-government subsidy strategy; meanwhile, the social welfare under these subsidy strategies increases with the increase of the spillover effects, and the vertical spillover effect has a more significant impact on social welfare than the horizontal spillover effect. The social welfare under the pharmaceutical enterprise innovation input subsidy strategy has a trend of firstly increase and then decrease. When the spillover effects are small, social welfare is higher than that under the non-government subsidy strategy; when the spillover effects are large, social welfare is lower than that under the non-government subsidy strategy.

## 7 Conclusion

Based on the comprehensive consideration of the horizontal spillover effects and the vertical spillover effects, this paper analyzed and compared the optimal decision of pharmaceutical production supply chain and government under five strategies: non-government subsidy strategy, pharmaceutical enterprise innovation input subsidy strategy, pharmaceutical enterprise innovative product subsidy strategy, patient price subsidy strategy and patient medical insurance subsidy strategy, which are shown as follows:

1. No matter what subsidy strategy the government adopts, it will have a positive incentive effect on the profits of pharmaceutical enterprises and social welfare. Among them, the most effective subsidy strategy is the patient medical insurance subsidy strategy, which can optimize the enterprises’ profits and social welfare. Meanwhile, there are two similar subsidy strategies: pharmaceutical enterprise innovative product subsidy strategy and patient price subsidy strategy, which will only change the pharmaceutical enterprises’ product pricing strategy, and other decision-making behaviors and profits are the same.
2. The horizontal spillover effects and the vertical spillover effects of new drug R&D have different impacts on decisions of enterprises and government. Except for the pharmaceutical enterprise innovation input subsidy strategy, regardless of the subsidy strategy adopted, pharmaceutical enterprise innovation input and profits, as well as social welfare will increase with the increase of double spillover effects, and the vertical spillover effects have more significant effect on decision variables and income than the horizontal spillover effects. Therefore, the increase in vertical spillover effects can bring greater profits and social welfare.
3. The patient medical insurance subsidy strategy is the optimal subsidy strategy, which can generate higher profits for pharmaceutical enterprises and higher social welfare. Besides, Under the pharmaceutical enterprise innovation input subsidy strategy, the impact of spillover effects on the decision-making and profits of enterprises are unique. When the spillover effects are small, the spillover effects have a positive effect on the innovation input and profits of pharmaceutical enterprises, and social welfare; when the spillover effects are large, as the spillover effects continue to increase, the innovation input and profits of enterprises as well as the society welfare tend to decrease. Thus, when adopting this subsidy strategy, the government should focus on the impact of spillover effects.

In practice, our work can bring inspiration to the government and pharmaceutical enterprises in new drug R&D. First of all, in order to improve the level of new drug R&D of pharmaceutical enterprises, government departments should promote enterprises to carry out new drug R&D through subsidy strategies, guide patients to purchase new drug, forming a virtuous circle and promoting the development of pharmaceutical industry. Secondly, the government should choose the patient medical insurance subsidy strategy from the perspective of social welfare. This is the most beneficial for pharmaceutical enterprises and patients. However, in reality, it is difficult for new drugs to be included in medical insurance and there are few new drugs successfully included in medical insurance. Therefore, when the government departments formulate the medical insurance catalogue, they should give more opportunities for new drugs. Finally, the positive incentive effect of spillover effects can bring new impetus to pharmaceutical production supply chain. Therefore, the upstream and downstream pharmaceutical enterprises should cooperate wholeheartedly while ensuring their own benefits, strengthen the technological synergy in new drug R&D, strengthen information and technology exchanges, continuously improve spillover levels, and make full use of spillover effects, especially the benefits of vertical spillover effects, and make decisions systematically.

There are still many shortcomings in this paper, which also provide directions for future research. Firstly, in order to simplify, this paper assumes that the horizontal spillover effects between enterprises in the industry is the same, and the vertical spillover effects between upstream and downstream enterprises is the same. These stricter assumptions need to be further relaxed and expanded in the future. Secondly, this paper only considers the independent R&D scenario of pharmaceutical enterprises, but in reality, pharmaceutical enterprises can carry out a variety of cooperation modes, which needs further study. Thirdly, there are technical risks in the R&D and innovation process of pharmaceutical enterprises, and the probability of success can be introduced into research in the future.

## Supporting information

S1 Appendix (DOCX)
