# Supplementary material for: Research on government subsidy strategies for new drug R&D considering spillover effects
Source: PLoS One. 2022 Feb 10;17(2):e0262655. doi: 10.1371/journal.pone.0262655 (PMC8830673; doi:10.1371/journal.pone.0262655)
Supplement: S1 Appendix — (DOCX) [file pone.0262655.s001.docx]

**S1 Appendix**

Proof of Lemma 1:

According to formulas (5) and (6), the profit functions of upstream and downstream pharmaceutical enterprises are as follows:

（A.1）

（A.2）

The upstream and downstream pharmaceutical enterprises pursue their own profit maximization, which are as follows:

（A.3）

（A.4）

First of all, in the product market, the upstream and downstream pharmaceutical enterprises determine the optimal output through the Cournot competition model under the government policy. take the derivative of $\pi_{di}^{B}$with respect to $q_{di}$, and set , then it is obtained as follows:

（A.5）

Bring (A.5) into $p=a-bQ$ to get:

（A.6）

In the same way, from the formula (A.5) can be obtained as follows:

（A.7）

Then, the upstream enterprise makes the optimal output decision. According to the formula (A.5) (A.6) (A.7), take the derivative of $\pi_{si}^{B}$with respect to $q_{si}$, and set , then it is obtained as follows:

（A.8）

According to the description of Assumption 1, the total output of upstream pharmaceutical enterprises and downstream pharmaceutical enterprises is equal,,then it is obtained as follows:

（A.9）

Bring (A.9) into $p=a-bQ$ to get:

（A.10）

Bring (A.8) into (A.7) ,then it is obtained as follows:

（A.11）

Upstream and downstream enterprises make R&D investment decisions, and all enterprises have their own profit maximization goals, which are as follows:

（A.12）

（A.13）

Take the derivative of $\pi_{si}^{B}$ and$\pi_{di}^{B}$ with respect to $x_{si}$ and $x_{di}$, and set , , then it is obtained as follows:

（A.14）

（A.15）

Finally, put (A.14) (A.15) into the above formula to get Lemma 1.
